# Ligand-Mediated Proton-Coupled Electron Injection into Reactive Cores of Soluble Macroanion-Like Complexes of Titanium Dioxide

**DOI:** 10.1021/jacs.5c05809

**Published:** 2025-07-07

**Authors:** Manoj Raula, Sapir Dabush Avnaim, Aranya Kar, Meital Samin, Shubasis Roy, Mark Baranov, Nitai Leffler, Zhong-Ling Lang, Josep M. Poblet, Ira A. Weinstock

**Affiliations:** † Department of Chemistry, 77282 Amity University Noida, Noida, Uttar Pradesh 201303, India; ‡ Department of Chemistry, 26732 Ben-Gurion University of the Negev, Beer Sheva 84105, Israel; § Ilse Katz Institute for Nanoscale Science & Technology, 26732 Ben-Gurion University of the Negev, Beer Sheva 84105, Israel; ∥ Key Laboratory of Polyoxometalate and Reticular Material Chemistry, Faculty of Chemistry, Northeast Normal University, Changchun 130024, China; ⊥ Departament de Química Física i Inorgànica, 16777 Universitat Rovira i Virgili, Tarragona 43007, Spain

## Abstract

Although widely utilized as reactive components of devices and functional materials, the harnessing of metal-oxide nanocrystals (NCs) as ligand-tunable reaction centers of soluble catalysts remains an ongoing challenge. We now report that readily modifiable, oxidatively inert polyoxometalate (POM) ligands amplify and rationally control rates of photocatalytic H_2_ evolution by anatase-TiO_2_ NCs. This was achieved using a series of four POM-derived oxo-donor ligands, [(X^n+^W_11_O_39_)­Ti^IV^–O^–^]^(10‑n)–^, X^n+^ = Al^3+^, Si^4+^, P^5+^, and [(P_2_W_17_O_61_)­Ti^IV^–O^–^]^8–^ (written here as oxo-anion donors) coordinated via bridging-oxo linkages to Ti atoms at the surfaces of 7 ± 1.6 nm anatase-TiO_2_ NCs, wherein the four respective macroanion-like complexes are each ligated, on average, by 90 ± 15 POM anions. Under UV–vis light irradiation in water (10% MeOH), rates of H_2_ evolution are a linear function of the quantum yield for photocatalytic oxidation of MeOH by the Ti-substituted POM ligands. This rate-limiting ligand reduction is followed by rapid visible-light driven proton-coupled electron injection into TiO_2_, which proceeds until rates of H_2_ evolution achieve parity with those of POM reduction, resulting finally in a dynamic steady state. Here, the NC core of the most reactive, [(P^5+^W_11_O_39_)­Ti^IV^–O−]^5–^−ligated complex, **3**, is populated by 440 electrons and 440 protons, representing an effective H^+^ concentration of 10 M (per nm^3^ of TiO_2_). The findings show that metal-oxide NCs can function as the reactive centers of ligand-tunable catalysts and, more generally, provide fundamental mechanistic understanding that establishes new structure–reactivity relationships for the design of rationally tunable modular components of functional materials.

## Graphical abstract

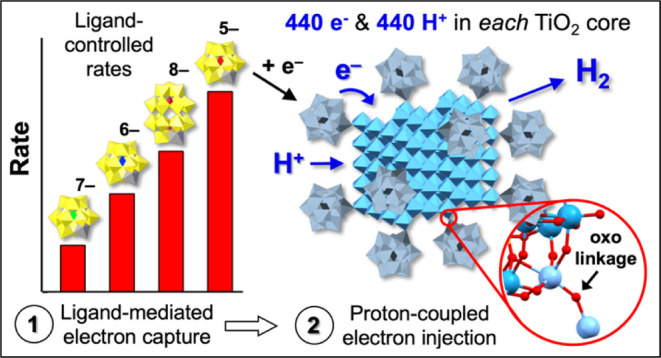

## Introduction

Applications of metal-oxide nanocrystals (NCs)^1–9^ are highly diverse, with catalytic, electrochemical, photochemical and electronic properties paralleling those of their solid-state analogs. And, although widely utilized as components of functional assemblies and solid-state materials, the harnessing of metal-oxide NCs as rationally tunable catalytic centers remains an ongoing challenge.

The main obstacle is that charge-stabilized colloidal metal-oxide NCs are highly susceptible to precipitation, especially in water.^10,11^ This was addressed by using solvothermal synthetic methods and organic ligands to confer stability in organic media. Although organic ligands provide remarkable control over size, shape and crystalline phase,^1,12^ they block access to reactive surfaces in catalysis, and despite exceptions,^13^ function as “insulating barriers”,^6,9,14^ preventing electronic coupling or the emergence of optoelectronic properties.^2,8^ Thus, to advance the use of NCs in the design of functional materials,^1,2,6,15,16^ ligand exchange^6,14,17^ and oxidative stripping^18^ methods were developed to remove the organic ligands and induce NC aggregation^5^ or to replace the organic ligands by small inorganic anions.

Until recently,^4,19–23^ however, solution-state catalysis by stable metal-oxide NCs was limited to specific cases^13^ or to water-stable colloids such as those of iridium oxide.^24^ Accordingly, the use of ligation to control catalysis by metal-oxide NCs remains a largely undeveloped topic within the science of soluble nanostructures.

In this regard, metal-oxide cluster anions (polyoxometalates, or POMs) form bridging-oxo linkages to metal atoms at the surfaces of phase-defined metal-oxide NC cores, resulting in isolable, water-soluble nanostructures uniquely positioned between molecular polyoxotitanate clusters^25,26^ and electrostatically stabilized colloids.^10^ The POM ligands feature oxidative stabilities unmatched by organic ligands or adsorbed sensitizers,^27^ and allow for substrate access by binding to a fraction of the metal atoms at complexed-NC surfaces. Moreover, POMs provide stable, water-soluble complexes of metal-oxide, oxyhydroxide and hydroxide NC cores, such as those of anatase-TiO_2_,^19^ α-Fe_2_O_3_ (hematite),^20,21^ monoclinic-CuO,^4^ γ-FeOOH (lepidocrocite),^22^ dzahlindite-In­(OH)_3_^23^ and 6-line ferrihydrite (approximately Fe_5_O_3_(OH)_9_),^28^ and of reactive “molecular nanoparticles” of spinel-Co_3_O_4_ ^29^ and cubic-NiO.^30^ Many POMs are also reversible electron acceptors^31^ with tunable redox potentials^32,33^ and readily modifiable photochemistries.^34,35^

We now show that the POM anions used to ligate water-soluble macroanion-like complexes of anatase-TiO_2_ NCs amplify rates of photocatalytic H_2_ evolution. Investigation of sequential reaction steps, including proton-coupled electron injection, shows how rates can be rationally controlled by incremental changes in the photophysical properties of the POM ligands.

## Results and Discussion

### A Series of POM-Complexed TiO_2_ NCs

A previously reported method^19^ for ligation of POMs oxo-donors to TiO_2_ NCs was used to give an expanded series of complexes, within which the reduction potentials of the ligating Keggin and Wells-Dawson derived anions span a range of over 0.5 V. The synthesis involves hydrolysis of titanium­(IV) isopropoxide in aqueous solutions of monolacunary Keggin anions [X^n+^W_11_O_39_]^(12‑n)–^, X^n+^ = P^5+^, Si^4+^, Al^3+^, and the monolacunary Wells-Dawson anion, [P_2_W_17_O_61_]^10–^, followed by heating at 170 °C for 24 h. The lacunary anions react with Ti to eventually serve as oxo-donor ligands, [(X^n+^W_11_O_39_)­Ti^IV^–O^–^]^(10‑n)–^ and [(P_2_W_17_O_61_)­Ti^IV^–O^–^] ^8^−, coordinated to Ti atoms at the surface of ca. 7 nm anatase-TiO_2_ NC cores (Figure 1). These reactions give optically transparent near-neutral solutions of the POM-ligated complexes (see the Supporting Information for experimental details and characterization data in Figures S1–S13).

**Figure 1:**
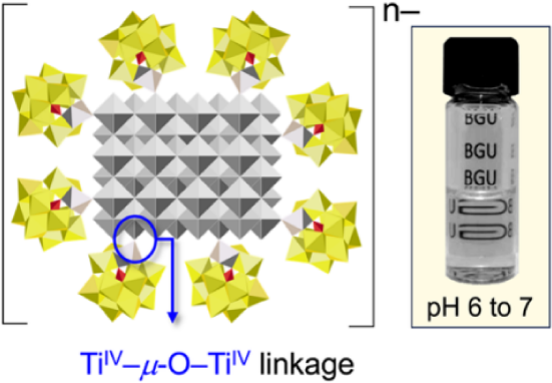
Ti­(IV)-substituted Keggin anions coordinated to the surface of a TiO_2_ NC via bridging μ-oxo linkages. At right is a representative optically transparent solution of these macroanion-like complexes.

The POM-complexed Ti^IV^ atoms reside in pentacoordinate lacunary binding sites and the formation of bridging-oxo linkages to Ti^IV^ atoms at the surfaces of TiO_2_ NCs (Figure 1) is structurally analogous to the documented condensation of POM-complexed Ti^IV^=O moieties (eq 1):^36–39^

1 $$2{\left[\left({\mathrm{PW}}_{11}O_{39}\right)Ti^{\mathrm{IV}}=O\right]}^{5-}+2H^{+}\to {\left[\left({\mathrm{PW}}_{11}O_{39}\right)Ti^{\mathrm{IV}}-\mu ‐O-Ti^{\mathrm{IV}}\left({\mathrm{PW}}_{11}O_{39}\right)\right]}^{8-}+H_{2}O$$

The four oxo-donor ligands used in this study are shown at the top of Figure 2. Cryo-TEM images of the corresponding POM-complexed NCs are provided at the bottom of Figure 2. Complexes of the Keggin-anion ligands with heteroatoms Al^3+^, Si^4+^, P^5+^ are labeled, **1**, **2** and **3**, respectively, and the Wells-Dawson (WD) anion complex is labeled **4**.

**Figure 2:**
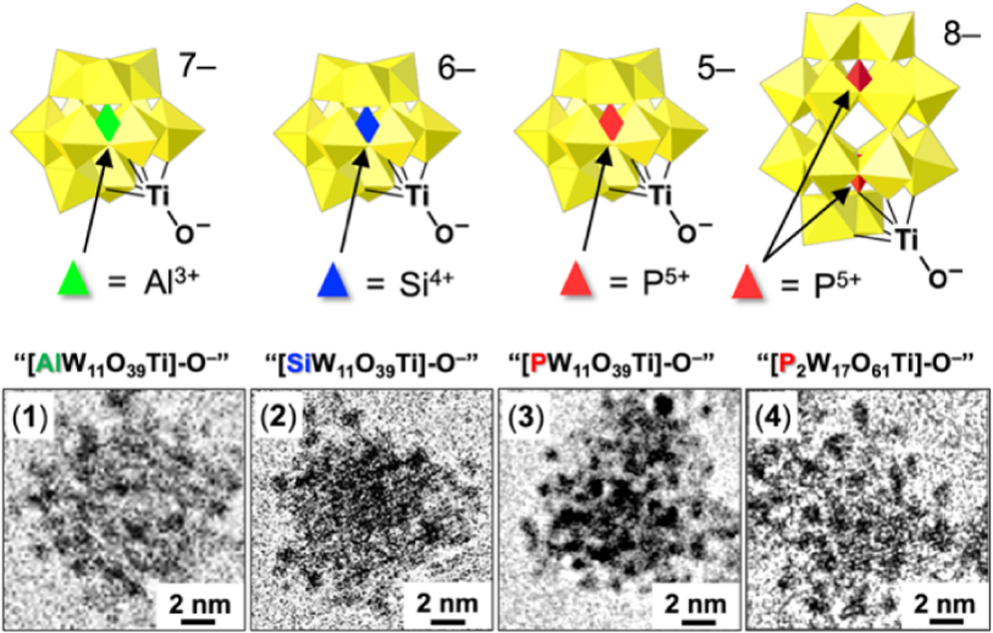
Ti^IV^-substituted Keggin and Wells–Dawson cluster-anion complexes of anatase-TiO_2_ NCs. At the top are structures and formulas of the four Ti^IV^-substituted POM ligands. Below these are cryo-TEM images of the corresponding TiO_2_-core complexes, **1**, **2**, **3**, and **4**, labeled at the upper-left of each image (see Figures S4). The numerous small dark objects on each TiO_2_ core are the POM ligands, which are readily imaged due to their electron-dense tungsten atoms (see Figures S6–S8) and identified by electrospray-ionization mass spectroscopy (ESI-MS; see Table 1, Figures S12 and S13).

Isolation, purification and full characterization of each complex, including confirmation of the anatase phase of TiO_2_, average TiO_2_–NC size, the structure and composition of the bound POM ligands, and the average number of POMs on each NC, were carried out using published methods (see Figures S5–S13).^4,19–23,28^ In particular, ESI-MS of acid-cleaved ligands (Figures S12 and S13) was used to confirm that the dark ca. 1 nm objects in the cryo-TEM images in Figure 2 are the indicated POM anions. The anatase phase and ca. 7 nm size of the TiO_2_ cores of all four complexes were confirmed by X-ray diffraction (XRD, Figure S3), TEM (Figure S5) and high-resolution TEM (HRTEM, Figures S6–S8). The estimated numbers of POM ligands on average-sized NC cores of each complex were determined after using energy-dispersive X-ray spectroscopy (EDX) to quantify W to Ti ratios (Figures S6–S8). These ratios indicated ligation by an average of 90 ± 15 POMs per NC. These findings are summarized in Table 1, while the fitting parameters used to estimate numbers of POM ligands per NC are provided in Figures S6–S8.

**Table 1:**
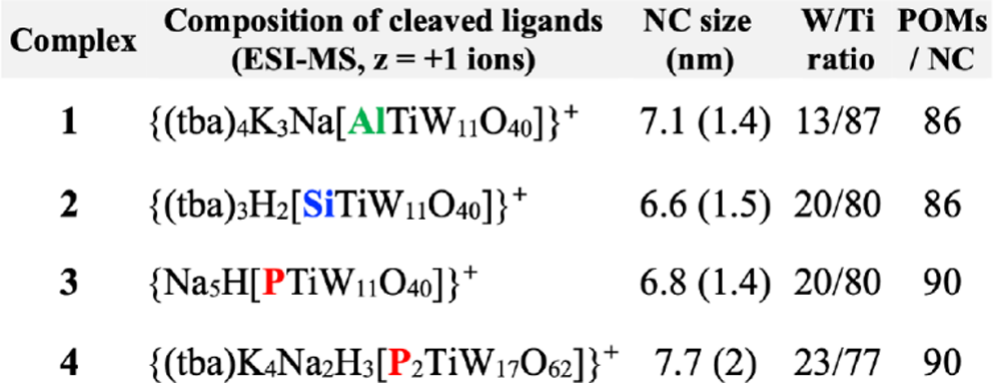
Ligand Characterization by ESI-MS,^a^ Mean TiO_2_ NC Sizes and Standard Deviations,^b^ W/Ti ratios,^c^ and Average Numbers of POM Ligands per TiO_2_ NC^d^

a tba is an abbreviation for *n*-Bu_4_N, added to isolate the acid-cleaved ligands prior to ESI-MS analysis.

b From histograms of TiO_2_ core sizes in TEM images; uncertainties are in parentheses.

c From EDX measurements.

d Average values required to fit calculated to observed W/Ti ratios for each mean NC size. Fitting to experimental W/Ti ratio values required varying the area allocated to each POM ligand on the TiO_2_ surface as follows: 2.5 nm^2^ for **1**, 1.80 nm^2^ for **2**, 1.81 nm^2^ for **3,** and 2.1 nm^2^ for **4**. Sensitivity analysis based on fitted surface-areas allocations and variations in NC dimensions suggested an uncertainty of ± 15 POM ligands per average-sized NC. Fitting parameters are provided in Figures S6–S8.

### Ligand-Controlled Hydrogen Evolution

Due to the solubility of the POM-complexed NCs and the abundance of POM ligands on each TiO_2_ core, it was possible to observe the first one-electron reductions of the coordinated ligands^19–21^ by differential-pulse voltammetry (DPV; 100 mM LiClO_4_ electrolyte; Figures 3a and S9). Reduction potentials of the Keggin-derived ligands move in the positive direction by ca. 150 mV per unit decrease in negative charge, and do so in a linear fashion as originally reported by Pope for plenary Keggin anions.^40^ The reduction potential of the coordinated Wells-Dawson (WD) anion^41^ is less negative than those of the Keggin ions. The potentiometric interrogation of ligands bound to metal-oxide NCs in solution has little precedent in the literature.^19–21^ In the present work, this information is used to understand the role of the ligands on the photochemical activity of the complexed TiO_2_ cores.

**Figure 3:**
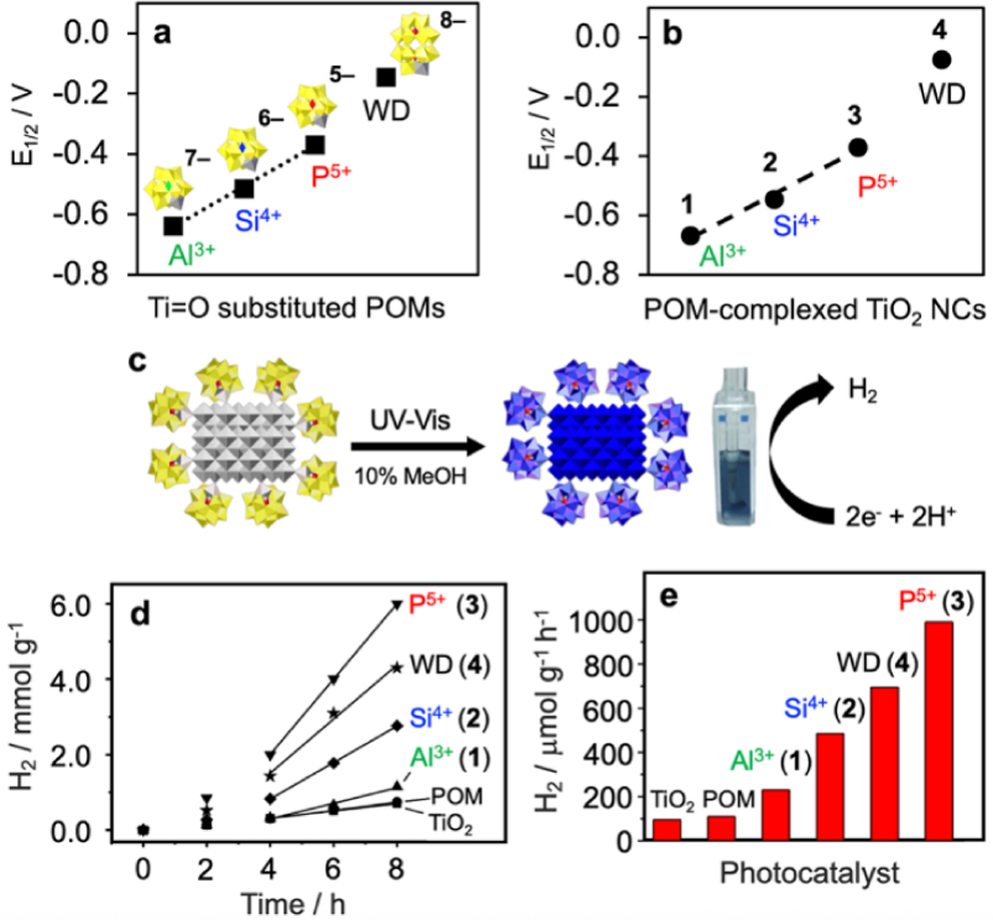
(**a**) Reduction potentials (by differential-pulse voltammetry, DPV) of the oxo-donor ligands, [(X^n+^W_11_O_39_)­Ti^IV^–O^–^]^(10‑n)–^, X^n+^ = P^5+^, Si^4+^, Al^3+^, and [(P_2_W_17_O_61_)­Ti^IV^–O^–^]^8–^ of complexes **1**-**4** (Figure S9) and (**b**) via cyclic voltammetry of molecular Ti^IV^=O substituted POM anions, [α-X^n+^Ti­(O)­W_11_O_39_]^(10‑n)–^ and [α_2_-P_2_Ti­(O)­W_17_O_61_]^8–^, deployed as models for the POM ligands in complexes **1**-**4** (Figure S10). (**c**) Photochemical H_2_ evolution using 10% MeOH under UV–vis irradiation (150 W Xe lamp), during which the solution turned blue, as shown at right. (**d**) H_2_ produced per g of TiO_2_ as a function of time for complexes **1**, **2**, **3** and **4**; smaller rates were observed in control experiments using commercial anatase-TiO_2_ NCs (labeled TiO_2_) and the Ti=O substituted Keggin anion, [α-PTi­(O)­W_11_O_39_]^5–^ (labeled POM), a close analog of the Ti-substituted phosphotungstate ligand in complex **3**. (**e**) Rates of H_2_ evolution. For complexes **1** - **4**, these were calculated from the slopes of the linear (R^2^ = 0.99) increases in H_2_ with time from 4 to 8 h in panel (d), i.e., after the initial induction period discussed later in the text. For the two control experiments, which did not exhibit induction periods, the rates refer to H_2_ evolved over 8 h.

To confirm these values (Figure 3a) indeed correspond to reduction of the four μ-O linked POM ligands, reduction potentials were determined for the corresponding series of molecular analogs: α-[X^n+^Ti­(O)­W_11_O_39_]^(10‑n)–^, X^n+^ = Al^3+^, Si^4+^, P^5+^, and α_2_-P_2_Ti­(O)­W_17_O_61_]^8–^, prepared as described in the SI. As shown in Figure 3b, the reduction potentials of the molecular analogs are similar to, and follow the same trend, as those observed for the TiO_2_-bound POMs (Figure 3a). As seen in Figure 3a, the reduction potentials of the POM ligands in the four complexes span ca. 0.5 V, from less than −0.6 V (vs NHE) for **1** to more positive than −0.2 V for **4**.

Because the activities of POMs involved in electron-transfer processes nearly always track with their reduction potentials and/or charge, it was reasonable to expect a similar situation would apply to the effects of the four POM ligands on the complexed TiO_2_ cores. As such, the reactivities of the four complexes were expected to increase or decrease as the ligands were varied in complexes **1** to **4**. This was evaluated for photochemical H_2_ evolution, carried out using 10% MeOH by volume in water under UV–vis irradiation (150 W Xe lamp) (Figure 3c). Notably, previous work^19^ showed complex **3** (P^5+^) to be substantially more reactive in H_2_ evolution than **2** (Si^4+^). Based on that result, the trend in reduction potentials of the four complexes (Figure 3a), led to the expectation that rates of H_2_ evolution would increase in the order: **1** < **2** < **3** < **4**. However, data provided below unexpectedly showed that **3** gave the fastest rate of H_2_ evolution.

H_2_ evolution data for the four complexes irradiated under identical conditions are provided in Figure 3d,e. The data in Figure 3d show H_2_ produced as a function of time per gram of TiO_2_. Also shown are control experiments under the same conditions carried out using commercial anatase-TiO_2_ NCs and the Ti=O substituted Keggin anion, α-[P^5+^Ti­(O)­W_11_O_39_]^5–^, a model for the Ti-substituted phosphotungstate ligand in the most reactive complex, **3**. Mechanistically important aspects of H_2_ evolution (Figure 3d) were induction periods of close to 4 h, followed by more rapid H_2_ evolution that proceeded linearly with time. The slopes of the rapid-phase plots are reported as rates in Figure 3e. Another mechanistically important observation was the lack of a correlation between the reduction potentials of the POM ligands (Figure 3a,b) and rates of H_2_ formation (Figure 3d,e). Moreover, no correlation was observed between rates and POM charge. That is, rates increased in the order **1** < **2** < **4** < **3**, while the charges of the respective POM ligands varied from 7- to 6- to 8- to 5-.

In addition, control experiments ruled out H_2_ formation by the photochemically active^34^ POM ligands themselves—rather than by TiO_2_—as the basis for the relative reactivities of the four complexes. This was determined by measuring rates of H_2_ formation using 10% MeOH solutions of molecular Ti^IV^=O substituted POM anions, [α-X^n+^Ti­(O)­W_11_O_39_]^(10‑n)–^, X^n+^ = Al^3+^, Si^4+^, P^5+^ and [α_2_-P_2_Ti­(O)­W_17_O_61_]^8–^, as models for the ligands on complexes **1**-**4** (Figure S14). Not only are rates of H_2_ evolution much smaller than those of complexes **1**-**4**, but [Si^4+^Ti­(O)­W_11_O_39_]^6–^ is the most active POM anion. This large deviation from the trends in Figure 3d,e ruled out direct H_2_ formation by the POM ligands as the basis for the observed order of reactivity and, in addition, confirmed that H_2_ evolution must occur at TiO_2_.

The basis for the unexpected order of reactivity of the four complexes (Figure 3d,e) was revealed by measuring the quantum yields of UV-driven photochemical oxidation of MeOH by molecular analogs of the four POM ligands. As originally noted by Darwent,^42^ quantum yields for Keggin anions do not track with their redox potentials. Quantum yields for MeOH oxidation were determining by using the method of Hatchard and Parker^43^ to quantify rates of reduction of the molecular analogs of the ligands in complexes **1–4**: [X^n+^Ti­(O)­W_11_O_39_]^(10‑n)–^, X^n+^ = Al^3+^, Si^4+^, P^5+^, and [P_2_Ti­(O)­W_17_O_60_]^8–^. Once done, a linear correlation was observed between rates of H_2_ evolution by complexed TiO_2_, which increased in the order: **1** < **2** < **4** < **3**, and the quantum yields for reduction of the molecular POM-ligand analogs (Figure 4).

**Figure 4:**
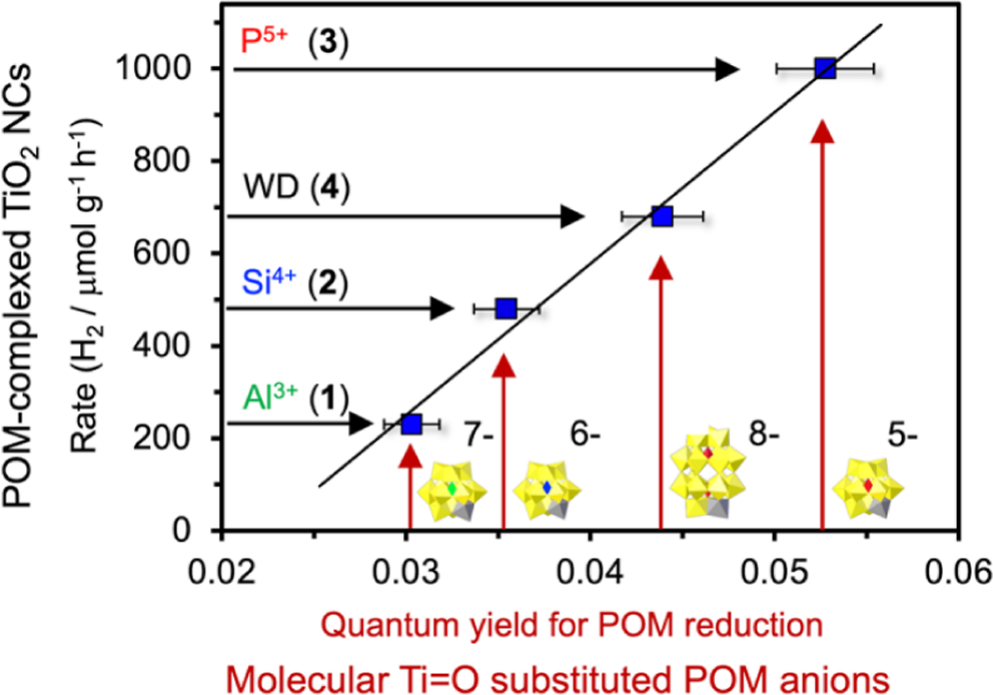
Rates of photocatalytic H_2_ evolution (150 W Xe lamp; 10% MeOH) by complexes **1**-**4** as a function of quantum yields at 280 nm for reduction of molecular Ti=O-substituted POM anions, [X^n+^Ti­(O)­W_11_O_39_]^(10‑n)–^ (X^n+^ = Al^3+^, Si^4+^, P^5+^) and [P_2_Ti­(O)­W_17_O_60_]^8–^, deployed as models for the respective μ-oxo coordinated POM ligands. The linear correlation coefficient is 0.99 and the error bars are set at ± 5% (see the Materials and Methods section of the SI for details).

This linear correlation identified photochemical reduction of the POM ligands as the rate limiting step in H_2_ evolution from TiO_2_. This finding led to two mechanistic questions: **1**) What drives electron transfer (ET) from reduced POM ligands to TiO_2_ and, **2**) What is responsible for the lag time before onset of rapid H_2_ evolution revealed in Figure 3d?

Even though H_2_ evolution was carried out under the unfiltered UV–vis spectrum provided by the Xe lamp, individual steps were found to require much less energy. The driving force for electron transfer from the reduced POM ligands to TiO_2_ was found to occur under visible light alone. This was first addressed experimentally (Figure S15). The optimal experiment would have been to selectively reduce the POM ligands on the complexed NCs and to then investigate rates of thermal, visible-light and UV-light driven ET to TiO_2_. However, it was found that TiO_2_ is reduced at the potentials required to reduce the POM ligands, so that selective ligand reduction could not be achieved. An alternative approach was then used, in which the one-electron-reduced form of the molecular analog, [PTi­(O)­W_11_O_39_]^5–^, of the POM ligands in complex **3** was added to a solution of the fully oxidized form of complex **3** itself, after which ET to the TiO_2_ core was quantified by UV–vis spectroscopy (Figure S15). While very little reduction of the TiO_2_ core was observed after 2 h in the dark at room temperature, the degree of reduction of the TiO_2_ core increased by a factor of ca. 70 after only six min of visible light irradiation (150 W Xe lamp with a 400 nm cutoff filter). Given the similar reduction potentials of [PTi­(O)­W_11_O_39_]^5–^ and the same cluster-anions, [(PW_11_O_39_)­Ti^IV^–O^–^]^5–^, bound via μ-oxo linkages to TiO_2_ (Figure 3a,b), outer-sphere ET from the reduced molecular anion, [PTi­(O)­W_11_O_39_]^6–^ to the bound anions, a process energetically similar to electron self-exchange, should occur rapidly.^31,33^ As such, the rapid visible-light driven reduction of TiO_2_ is most likely due to electron injection from the reduced POM ligands.

Given the assumptions required to interpret the result of that experiment, verification was sought through computational methods. For this, the ligating-POM anion, reduced by one electron, i.e., [(PW^V^W^VI^_10_O_39_)­Ti^IV^–O^–^]^6–^, was coordinated to a (TiO_2_)_38_ fragment and the effects of irradiation at 622 nm were calculated (Figure 5; see details in Figures S16-–19 and Table S1).

**Figure 5:**
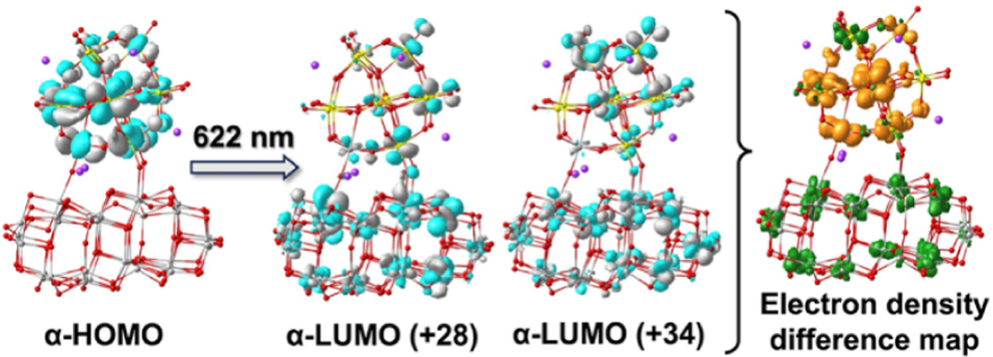
Molecular orbitals and electron density difference representations involved in the 622 nm transition for Na_6_PW_11_TiO_40_-(TiO_2_)_38_, a model for complex **3** with the POM ligand reduced by one electron. The highest occupied molecular orbitals containing the added electron are shown at left, and the two excitations in the center show that the 622 nm transition induces metal-to-metal charge transfer (MMCT) from W­(d) orbitals of the reduced POM to Ti­(3d) orbitals of TiO_2_. The electron density difference map at far right highlights regions where electron density increases in the TiO_2_ fragment (green) and decreases in the POM ligand (orange).

For the one-electron reduced POM ligand on the (TiO_2_)_38_ cluster, an absorption band at 622 nm (the S_0_ to S_38_ transition in Table S1) mainly arises from the α-HOMO to α-LUMO+28 and α-LUMO+34 excitations shown in Figure 5 (excerpted from Figure S19). Those unoccupied states show major donations from Ti­(3d), and relatively smaller contributions from the W­(5d) and O­(2p) orbitals of the POM. This character, indicating a metal-to-metal charge transfer (MMCT) mechanism wherein electrons transfer from the W­(4d) orbital of the POM to the Ti­(3d) of TiO_2_, would potentially occur under visible light irradiation, resulting in the reduction of TiO_2_. Moreover, the mixing of POM and TiO_2_ states at the interface suggests that the POM excited states are strongly coupled to TiO_2_, which may give rise to large rates of electron injection. Finally, electron transfer from W orbitals to TiO_2_ upon visible excitation^44^ is clearly seen in the electron-density difference map at the right in Figure 5.

Hence, the DFT simulations reveal that the reduced POM ligands improve the adsorption of TiO_2_ in the visible region and support rapid visible-light driven electron transfer from reduced POM ligands to TiO_2_. Notably, because rates of H_2_ evolution are directly correlated to quantum yields for POM oxidation of MeOH, the light-driven electron injection from reduced POM ligands to TiO_2_ is a “fast” step.

This rapid electron injection leads to an unprecedented degree of TiO_2_ reduction, with the uptake of an equivalent number of protons.^45^ TiO_2_ reduction was quantified by combined UV–vis spectroscopy and redox titration (Figure 6a,b), while proton uptake (Figure 6c) is discussed farther below.

**Figure 6:**
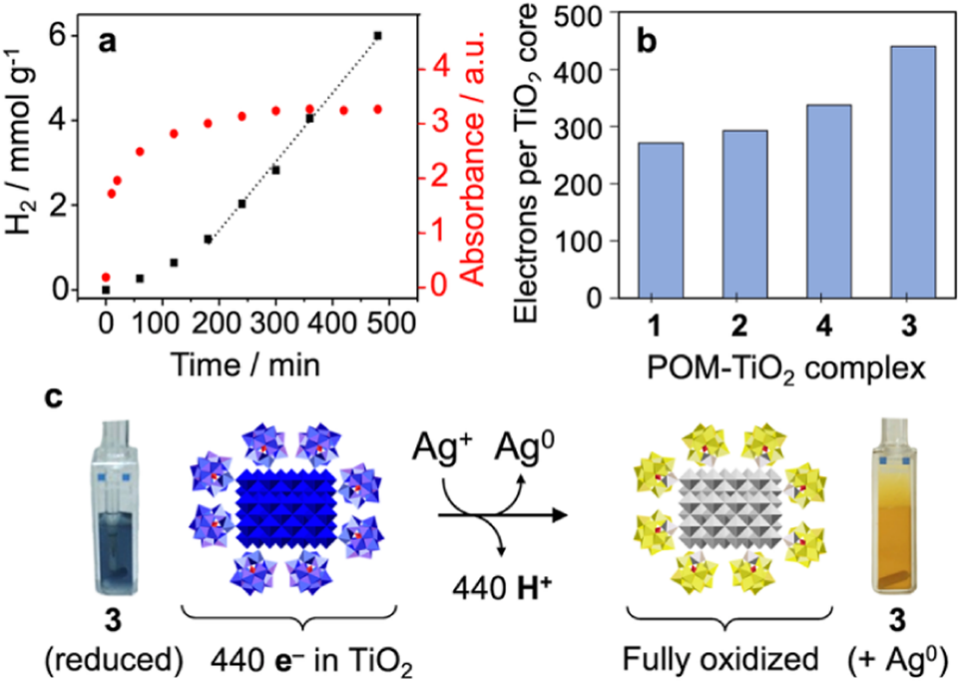
(**a**) Correlation between reduction of the TiO_2_ core of **3**, as indicated by absorbance at 640 nm (red dots; right-side *y* axis), and H_2_ evolution (black squares; left-side *y* axis). Notably, the degree of TiO_2_ reduction reaches a steady state after ca. 3–4 h of irradiation, as the onset of rapid H_2_ evolution prevents further increase in the degree of reduction of the TiO_2_ core (data for the other three complexes are provided in Figure S20). (**b**). Numbers of electrons in the TiO_2_ cores of the four complexes during steady-state turnover, reached after 4 h of irradiation (see Figure 3d). Uncertainties are estimated at ± 10%. (**c**) Electron-transfer to Ag^+^ of the 440 electrons present in the TiO_2_ core of each NC of **3** under steady-state turnover conditions (panel **b**), results in the proton-coupled releases of an equivalent number of protons (Figures S22–S23). Notably, the value of 440 electrons written below the reduced complex, shown in blue in (**c**) refers to the electrons in the NC core after subtraction of 1.6 electrons per each of the ca. 90 POM ligands of **3** under turnover conditions.

Figure 6a (red dots and right-side *y* axis) shows the absorbance of **3** at 640 nm in conjunction with the amounts of H_2_ produced under the same photoirradiation conditions (black squares and left-side *y* axis). Notably, the absorbance at 640 nm is predominantly due to electrons in TiO_2_ (see Figures S20 and S21), with some contribution from electrons in the reduced POM ligands, as discussed in conjunction with Figure 6b. The absorbance reaches a plateau after ca. 200 min and remains unchanged after 480 min (8 h). Meanwhile, H_2_ production proceeds slowly at first, and then at a more rapid and constant rate (Figure 6a, black squares). Notably, the onset of more rapid H_2_ production corresponds to the plateau phase of absorbance, indicative of a dynamic steady state. As such, the absorbance value at the plateau indicates the degree of TiO_2_ reduction at which the rate of H_2_ formation has increased sufficiently to reach parity with the rate of oxidation of MeOH by the [(PW_11_O_39_)­Ti^IV^–O^–^][Bibr ref5] ^-^ donor ligands of **3**. At this point, MeOH oxidation becomes rate limiting and as such, the rate of H_2_ evolution then remains constant. Absorbance vs time data for complexes **1**, **2**, and **4** are provided in Figure S20.

The numbers of electrons present in the TiO_2_ cores of complexes **1**-**4** during steady-state H_2_ production, Figure 6b, were obtained by redox titration with Na_2_Cr_2_O_7_ (Figure S21) and plotted after adjusting for numbers of electrons in the reduced POM ligands. The numbers of electrons in each ligand under turnover conditions were set equal to the numbers of electrons determined by dichromate titration of the molecular analogs of the bound ligands, reduced using MeOH and UV–vis light as shown in Figure S14.

The numbers of electrons in the TiO_2_ cores increase from **1** to **2** to **4** to **3**, the same order as that found for H_2_ evolution rates (Figure 3e) and of quantum yields for MeOH oxidation by molecular analogs of the POM ligands of the four complexes (Figure 4). For complex **3**, the ca. 440 ± 40 electrons in each NC core (Figure 6b), which on average contain ca. 4000 Ti atoms, represents a 10.8% reduction of Ti­(IV) to Ti­(III). This degree of photochemical TiO_2_ NC reduction is at least twice that of the largest value reported in the literature.^45–47^ While oxygen-depleted “black” titanium, TiO_*x*_,^48–50^ contains larger percentages of Ti­(III), the herein reported values are, to our knowledge, unprecedented for the photochemical reduction of anatase-TiO_2_.

The data in Figure 6a also explain the initially slow phase seen in Figure 3d, which is now assigned to charging of the TiO_2_ cores via electron injection by the POM ligands. During this phase, the degree of reduction of the TiO_2_ cores increases until the rate of H_2_ evolution reaches parity with that of the quantum-yield controlled reduction of the POM ligands (Figure 4). That parity defines the maximum (steady-state) rates of H_2_ evolution attained after 3 h of irradiation (Figure 3d), at which H_2_ formation is faster than rate-limiting reduction of the POM ligands.

The relatively rapid rate of H_2_ evolution is facilitated by extensive protonation of the highly reduced TiO_2_ cores. Namely, the 440 ± 40 electrons in each TiO_2_ NC of **3** under steady-state turnover conditions are charge balanced by the presence of an equal number of H^+^ ions.^45^ This was determined from the decrease in pH of a solution of highly reduced **3** upon complete proton-coupled electron-transfer oxidation by Ag^+^ (Figures 6c, see S22 and S23 and related discussion). The large number of protons in each NC gives an “effective” H^+^ concentration of 10 M in terms of protons per nm^3^ of TiO_2_. While the protons are not actually solvated, this effective concentration helps visualize the situation wherein an unprecedented concentration of electrons and protons reside within a single anatase-TiO_2_ NC. This results in H_2_ formation rates significantly faster than for colloidal TiO_2_, for which photochemical H_2_ evolution is slow in the absence of cocatalysts.^51^

Experimental evidence for the facile reaction of electrons with protons within highly reduced TiO_2_ was provided by the observation that, once **3** has been substantially reduced by 2 h of UV–vis irradiation, giving 1.8 mmol g^–1^ of H_2_ (Figure 3d), the material is sufficiently reactive that an additional 0.65 mmol g^–1^ of H_2_ is generated by visible light alone (395 nm cutoff filter; Figures S24– and S25). No additional H_2_ was observed when the sample was kept in the dark after 2 h of irradiation. Due to the absence of UV-driven photooxidation of MeOH by the POM ligands, the 0.65 mmol g^–1^ of H_2_ was a stoichiometric product, generated without additional proton-coupled electron injection into the TiO_2_ cores. As such, H_2_ evolution ceased when the numbers of electrons in each NC, quantified by UV–vis spectroscopy, decreased by ca. 35% of their initial values.

The above processes sum to a sequence of light-driven steps whose overall rate is controlled by the photochemical properties of the redox-active POM ligands, with **3** used as an example in Figure 7. First, the reduction potential of the TiO_2_ cores (Figure 7a) is estimated from published data^45^ to be substantially more negative than that of the POM ligands of **3** (Figure 3b). This is consistent with our finding that proton-coupled electron injection from reduced POM ligands to TiO_2_ is photochemically driven, even by visible light (>400 nm), while in the dark very little ET occurs (Figure S15). Computational data in Figure 5 indicate injection can be driven by 622 nm light, which is equivalent to 1.99 eV, much larger than the estimated 0.25 eV associated with the difference in reduction potentials shown in Figure 7a. This light-driven process involves the injection of ground-state electrons present in the reduced POM ligands after quenching by MeOH.

**Figure 7:**
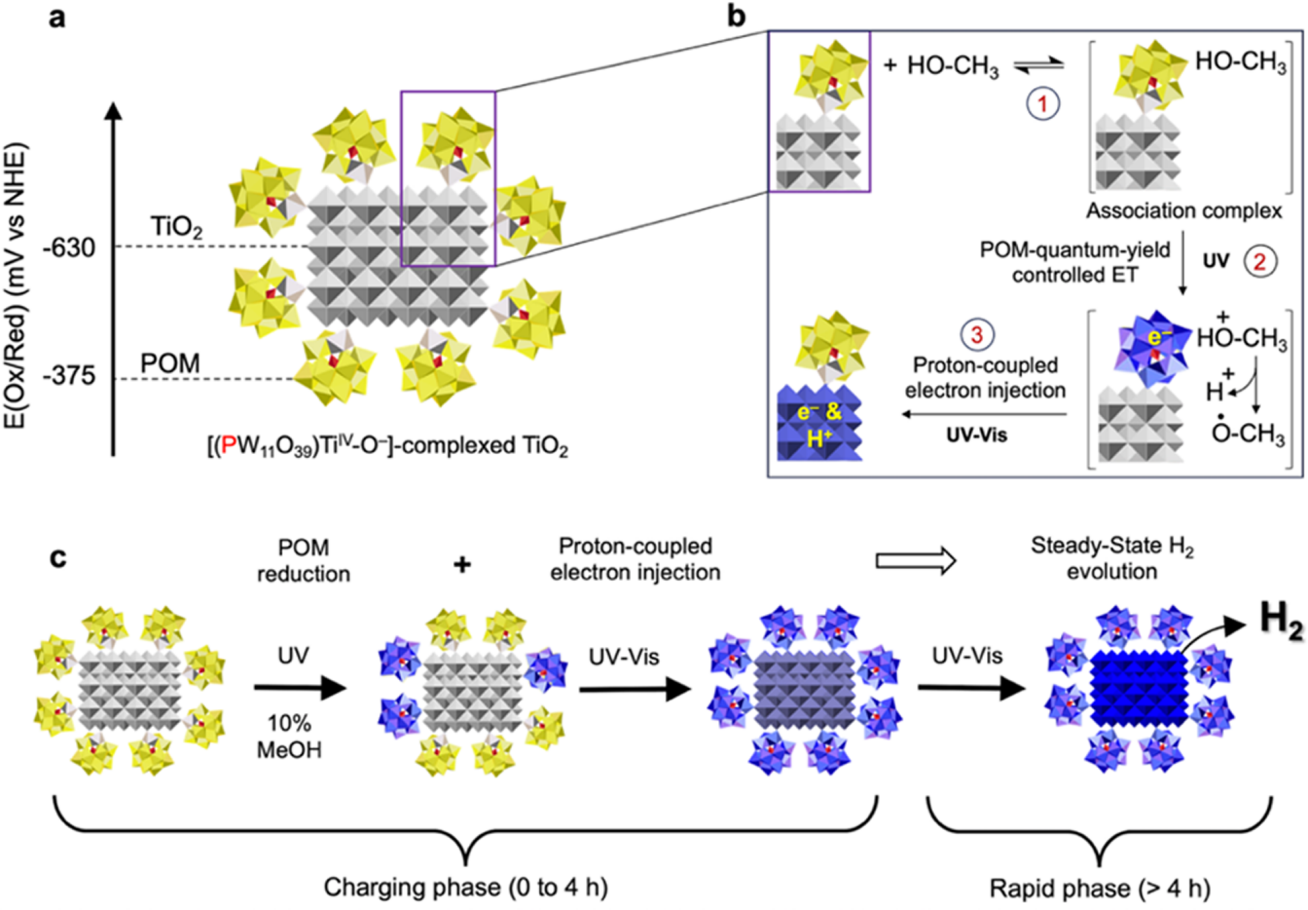
Light-driven steps that sum to the ligand quantum-yield controlled formation of H_2_. (**a**) Reduction potential of the [(PW^VI^_11_O_39_)­Ti^IV^–O^–^]^5–^ ligands and approximate reduction potential of the TiO_2_ core of **3**. (**b**) Transfer of electrons from MeOH to TiO_2_ is understood to proceed via three sequential steps, (1) MeOH association, (2) UV light driven ET from the [POM,MeOH] association complex to the POM ligand, and (3) proton-coupled electron injection into TiO_2_. (**c**) Charging of the TiO_2_ core leading to rapid, steady-state H_2_ evolution.

An alternative possibility is that short-lived photoexcited electrons in the POM ligands are trapped by TiO_2_ prior to reductive quenching by MeOH. However, available data argue that reductive quenching occurs prior to electron injection, with the passage of electrons from MeOH to TiO_2_ proceeding via the three sequential steps in Figure 7b.

The first two steps, 1 and 2, are based on mechanistic data by Fox,^52^ who reported that photochemical MeOH reduction of heteropolytungstate anions closely related to the heteropolytungstate anion ligands in compounds **1** to **3**, requires preassociation of MeOH to oxo groups of the POMs. She finds that, “*Photoexcitation of the photocatalyst:alcohol complex··· generates an excited state in which electron transfer from the adsorbed alcohol to the tungstate produces a reduced metal (W­(V)) complexed to the oxidized alcohol.*”^52^ Notably, this mechanism is particular to heteropolytungstate anions, and differs from that found for the decatungstate.^53^ Fox proposes that the next step in alcohol oxidation involves deprotonation of the organic radical cation. While not investigated in the present study, deprotonation of the MeOH radical cation is included by analogy in step 2 of Figure 7b.

Electron transfer within the photoexcited [POM,MeOH] association complex argues against the trapping of excited POM electrons by TiO_2_ prior to quenching by MeOH. This means that step 3 in Figure 7b should occur via photoexcitation of ground-state electrons within the W-based LUMO of the reduced POM ligands as shown in Figure 5. Additional evidence for POM reduction by MeOH prior to electron injection into TiO_2_ is suggested by the observation that H_2_ evolution rates for complexes **1** - **4** track with the quantum yields for UV-light driven oxidation of MeOH by molecular analogs of the ligands on the four different complexes (Figure 4). This correlation implies that the photophysics of the ligands themselves control their reduction by MeOH. If excited electrons in the POM ligands were injected into TiO_2_ prior to reduction of the POMs by MeOH, the injection process itself would “trap” the excited electrons, leading to large charge-separation lifetimes. It seems unlikely that these enhanced lifetimes would result in rates of ligand reduction by MeOH that precisely reproduce the rate-limiting quantum yields of the molecular analogs of the four ligands. In summary, light-driven electron injection into TiO_2_ likely occurs after reduction of the POM ligands by MeOH, as suggested by the three sequential steps in Figure 7b.

The charging and rapid phases in Figure 7c refer to the H_2_ evolution data in Figure 3d, where the reaction is slow during the first 2 h, and after a transition period, proceeds rapidly from 4 h onward. The first step in the reaction, indicated by the data in Figure 4, is reduction of the POM ligands via UV-light driven oxidation of MeOH. Experimental and computational data, Figure 5, show that the electron in the reduced POM ligand is coupled to Ti-based 3d orbitals in TiO_2_ such that even visible light can drive electron injection from the reduced ligands into TiO_2_. As illustrated in Figure 6c, ET to TiO_2_ is accompanied by proton uptake, and this proton-coupled electron injection continues until the rate of H_2_ formation becomes sufficiently rapid that the quantum-yield controlled reduction of the POM ligands becomes rate limiting, resulting in steady-state H_2_ evolution (Figure 3d). For complex **3**, this steady-state is characterized by the continuous presence and regeneration of 440 electrons and 440 proton within the TiO_2_–NC cores.

## Conclusions

Although widely utilized as reactive components of devices and functional solid-state materials, the harnessing of metal-oxide NCs as ligand-tunable catalytic centers remains an ongoing challenge. This is the context for the findings reported here, wherein POM complexation is used to rationally control the reactivities of anatase-TiO_2_ NCs via ligand-mediated proton-coupled electron injection. This was accomplished using a series of POM-derived oxo-donor ligands, [(X^n+^W_11_O_39_)­Ti^IV^–O^–^]^(10‑n)–^, X^n+^ = Al^3+^, Si^4+^, P^5+^, and [(P_2_W_17_O_61_)­Ti^IV^–O^–^]^8–^, coordinated via bridging-oxo linkages to Ti atoms at the surfaces of anatase-TiO_2_ NC cores (Figures 1 and 2). By design, the reduction potentials of the ligating POM anions spanned more than 0.5 V (Figure 3b), and reactivity in H_2_ evolution was initially expected to track with this parameter. Surprisingly, no correlation with POM reduction potential was observed. Rather, a linear correlation was found between rates of H_2_ evolution and the quantum yield for UV-light driven photocatalytic oxidation of MeOH by the Ti-substituted ligands (Figure 4). This identified POM reduction as the rate-limiting step and guided subsequent studies of visible-light driven proton-coupled electron injection from the reduced ligands into TiO_2_ (Figure 5). After several hours of reduction, the TiO_2_ cores become highly reduced (Figure 6), with the core of the most reactive complex, **3**, populated by 440 electrons and an equal number of protons. This 10.8% reduction of TiO_2_ is at least twice that of the largest reported value for photochemical reductions of anatase-TiO_2_.^45–47^ The equal number of protons gives an effective H^+^ concentration of 10 M in terms of protons per nm^3^ of TiO_2_. As a result, rates of H_2_ formation are significantly more rapid than for colloidal TiO_2_, for which photochemical H_2_ evolution is slow in the absence of cocatalysts.^51^ In the present case, percent-reduction of the TiO_2_ cores increases until H_2_ evolution becomes sufficiently rapid that photochemical oxidation of MeOH by the POM ligands becomes rate limiting, resulting in a dynamic steady-state (Figures 3d and 6a). Because the [(PW_11_O_39_)­Ti^IV^–O^–^]^5–^ ligands of complex **3** possess the largest quantum yield for MeOH oxidation of all four complexes studied (Figure 4), they serve as the most efficient “electron collectors” and, via rapid light-driven proton-coupled electron injection (Figure 5), generate the most highly reduced TiO_2_ cores (440 electrons and 440 proton, Figure 6) and the largest steady-state rate of H_2_ evolution (Figure 2e).

These findings show that metal-oxide NCs can indeed serve as the reactive centers of ligand-tunable catalysts. Importantly, the solubility and stability of these complexes made it possible to investigate their reaction mechanisms using the toolbox of solution-state methods typically reserved for molecular catalysis. This feature is used here to provide fundamental mechanistic understanding that establishes new structure–reactivity relationships for the design of rationally tunable modular components of functional materials.

## Supplementary Material



## Notes

Detailed experimental procedures, characterization methods, and data, DFT calculations, and additional figures and tables (PDF)
