# Supplementary material for: Ligand-Mediated Proton-Coupled Electron Injection into Reactive Cores of Soluble Macroanion-Like Complexes of Titanium Dioxide
Source: J Am Chem Soc. 2025 Jul 7;147(28):24653–61. doi: 10.1021/jacs.5c05809 (PMC12272697; doi:10.1021/jacs.5c05809)
Supplement: Supplementary file 1 [file ja5c05809_si_001.pdf]

Supporting information for:

Ligand Mediated Proton-Coupled Electron Injection into Reactive Cores of Soluble Macroanion-Like Complexes of Titanium Dioxide

Manoj Raula,[a] Sapir Avnaim,[b] Aranya Kar,[b] Meital Samin,[b] Shubasis Roy,[b] Mark Baranov,[c] Nitai Leffler,[b] Zhong-Ling Lang,[d] Josep M. Poblet,[e] Ira A. Weinstock[b,c]*

[a] Department of Chemistry, Amity University Noida, Uttar Pradesh 201303, India.
[b]Department of Chemistry, Ben-Gurion University of the Negev, Beer Sheva 84105, Israel.
[c]Ilse Katz Institute for Nanoscale Science & Technology, Ben-Gurion University of the Negev, Beer Sheva 84105, Israel
[d] Key Laboratory of Polyoxometalate and Reticular Material Chemistry, Faculty of Chemistry, Northeast Normal University, Changchun 130024, China.
[e]Departament de Química Física i Inorgànica, Universitat Rovira i Virgili, Tarragona 43007, Spain.
*Email: iraw@bgu.ac.il

Contents

Materials and methods

Figure S1. NMR spectra obtained after reaction of amorphous TiO_2(s) with all four lacunary POMs.

Figure S2. Dynamic light scattering (DLS) data before and after dialysis and after four cycles of precipitation and re-dissolution in water.

Figure S3. Powder X-ray diffraction of the anatase-TiO_2 nanocrystal (NC) cores.

Figure S4. Bright-Field-and additional cryo-TEM images of all four POM-ligated complexes of TiO_2

Figure S5. Histograms of the particle size in diameter and the average particle sizes of POM-TiO_2 complexes.

Figure S6. Bright field, HRTEM images and EDS analysis of the anatase-TiO_2 nanocrystal cores ligated by $[AlTiW_{11}O_{40}]^{7-}$(complex **1**).

Figure S7. Bright field, HRTEM images and EDS analysis of the anatase-TiO_2 nanocrystal cores ligated by $[SiTiW_{11}O_{40}]^{6-}$(complex **2**).

Figure S8. Bright field, HRTEM images and EDS analysis of the anatase-TiO_2 nanocrystal cores ligated by $[P_2TiW_{17}O_{62}]^{8-}$(complex **4**).

Figure S9. The differential pulse voltammograms (DPVs) of POM complexed TiO_2 NCs, **1**, **2**, **3** and **4**.

Table S10. The differential pulse voltammograms (DPVs) of the molecular Ti-substituted POMs used as analogs for those coordinated to TiO_2 NCs in complexes, **1**, **2**, **3** and **4**.

Figure S11. FTIR spectra of molecular POM anions used as ligands and baseline-corrected spectra obtained from POM-complexed TiO_2 NCs.

Figure S12. ESI mass spectra obtained after digesting complexes **1** and **2**.

Figure S13 ESI mass spectrum obtained after digesting the Wells-Dawson ligated complexe, **4**.

Figure S14. Rates of photocatalytic H_2 production by Ti-substituted POMs, used as models for their ligated analogs.

Figure S15. Experimental evidence for visible-light electron injection from reduced POMs into TiO_2.

Figure S16. DFT-determined structures of $Na_5PW_{11}TiO_{40}$ and $Na_6PW_{11}TiO_{40}$ ligands bound via bridging oxo linkages to a $(TiO_2)_{38}$ cluster.

Figure S17. Comparison of the simulated UV-visible spectra obtained using a B3LYP functional.

Table S1. Calculated excitation energy (E, eV), wavelength (λ, nm), oscillator strength (*f*) and composition of some representative transitions.

Figure S18. Molecular orbitals (isovalue = 0.02) and electron density difference (EDD) arising from visible-light driven HOMO to LUMO transitions from reduced $[PW_{11}TiO_{40}]^{n-}$ ligands to TiO_2.

Figure S19. Molecular orbitals and electron density difference representations involved in the S_{38} and S_7 transitions for $(TiO_2)_{38}$-$Na_6PW_{11}TiO_{40}$ (data from here are in Figure 5 of the text).

Figure S20. Absorbance vs. time data for TiO_2 complexes of **1**, **2**, and **4**.

Figure S21. Redox titration curve of the TiO_2-$PTiW_{11}O_{40}$ complex, **3**.

Figure S22. Redox titration of reduced complex **3** via oxidation by silver nitrate ($AgNO_3$).

Figure S23. Calculation of protons released during the oxidation of complex **3**.

Figure S24. Visible-light driven H_2 production from highly reduced NC cores.

Figure S25. Control experiment showing that H_2 production from highly reduced NC cores does not occur in the dark.

Materials and methods

Materials. Titanium(IV) tetra-isopropoxide (TTIP, 99.999%, Sigma-Aldrich, USA), isopropanol (Reagent Grade, Carlo Erba, France), NaCl (analytical grade, Frutarom, Israel), deuterium oxide (99.9% D, Cambridge Isotope Laboratories, UK), titanium(IV) dioxide (99% anatase, Alfa-Aesar), tetrabutylammonium bromide (TBABr, ACS reagent, ≥ 98%, Sigma-Aldrich, USA), acetonitrile (99.8%, Alfa-Aesar) HCl (analytical grade, Bio Lab, Ltd., Israel) and H_2O_2 (30 v/v%, Bio Lab, Ltd., Israel) were used as received. Sodium tungstate dihydrate ($Na_2WO_4{\cdot}H_2O$, extra pure) was purchased from Merck. Additional reagent-grade salts, acids and diethyl ether for polyoxometalate synthesis and reactions were obtained from commercial sources and used as received. Regenerated-cellulose dialysis membranes (45-mm flat-width tubes; 12−14000 Da MWCO) were purchased from VWR Scientific, treated before use to remove glycerin and traces of sulfur compounds, and stored in water at 5 °C. All water used for cleaning, synthesis and reactions was of high purity (18 MΩ resistivity) from a Millipore Direct-Q water-purification system. $Na_7[\alpha\text{-}PW_{11}O_{39}]{\cdot}12H_2O$,[1, 2] $Na_5[\alpha\text{-}PTiW_{11}O_{40}]{\cdot}xH_2O$,[3] $K_8[\alpha\text{-}SiW_{11}O_{39}]{\cdot}13H_2O$,[4] $K_9[\alpha\text{-}AlW_{11}O_{39}]{\cdot}13H_2O$,[5] $K_{10}[\alpha_2\text{-}P_2W_{17}O_{61}]{\cdot}20H_2O$[6] and $H_5[\alpha\text{-}AlW_{12}O_{40}]{\cdot}12H_2O$[5] were prepared by literature methods and checked by FTIR and ^{31}P, ^{27}Al and ^{29}Si NMR spectroscopy.

Instrumentation. General. pH values were measured using a EuTech pH 510 Bench-Top pH meter. UV-vis spectra were acquired using a HP 8452A spectrophotometer equipped with a diode-array detector (190-1100 nm range). FTIR spectra were acquired from KBr pellets using a Nicolet Impact 410 spectrophotometer. Al-27 and ^{31}P NMR spectra were acquired on a Bruker 400 MHz instrument, and ^{29}Si NMR spectra were acquired on a Bruker 500 MHz instrument. Chemical-shift values were externally referenced, respectively, to 0.10 M $AlCl_3$ ($[Al(H_2O)_6]^{3+}$), 1.0 M H_3PO_4 and tetramethylsilane (TMS), all set to $\delta = 0$ ppm, with internal lock signals tuned using D_2O. NMR spectral data were processed using Mnova version 5.1 (Mestrelab Research). Zeta potentials were measured using a ZEM 3600 Zetasizer (Malvern Instruments, Ltd). XRD data were obtained using a Panalytical Empyrean instrument using Cu K_α radiation (λ=1.5405 Å), operated at 40 kV and 30 mA, and equipped with a position sensitive (PSD) X'Celerator detector. DLS data was collected at 25 °C on an ALV-CGS-8F instrument (ALV-GmbH, Germany) at 90 deg (unless otherwise indicated), and the CONTIN method was used to obtain hydrodynamic radii (R_h). Prior to each measurement, solutions were filtered through 0.45 and/or 0.22 μm polyvinylidene fluoride (PVDF) Millipore filters. Electrospray ionization mass spectra (ESI-MS) were recorded from a LTQ Orbitrap XL instrument (Thermo Scientific, with an accuracy of 0.1 amu) with a nanospray ion source. TBA-salt of the POMs were used to make a solution in pure acetonitrile and directly injected for the ESI-MS measurements. Evolved hydrogen (photocatalysis studies) was quantified using a Thermo Scientific Focus Gas Chromatograph (GC) to analyze headspace gases sampled via a gas-tight syringe from septa-sealed reaction vessels. The GC was equipped with a dedicated thermal-conductivity detector (TCD), Ar was used as the carrier gas, and peak areas were calibrated using Scotty gas calibration standards (1% H_2 in N_2; Restek Corp).

Electron microscopy. Samples for transmission electron microscopy (TEM) and high-resolution TEM (HRTEM) were prepared by pipetting 5-10 μL of sample solutions onto carbon-coated Cu grids, and then allowing them to dry in air. In some cases, an SPS Spin150 spin coater was used to improve surface dispersion of solutes. TEM data were obtained using a FEI Tecnai 12 G2 electron microscope (120kV) equipped with a Gatan slow-scan camera.

Cryogenic-TEM. Samples for cryo-TEM imaging were prepared using a fully automated vitrification device ("Vitrobot"). First, 2.5-3 μL of the sample solution was placed by pipette onto a glow-discharged 300 Mesh Cu grid covered with a lacey-carbon film (Ted Pella, 01883-F) held inside a 100% humidity chamber. The grid was then mechanically "blotted" and immediately plunged into liquid ethane (b.p. 185K) cooled by liquid nitrogen (b.p. 77K). Data were collected on the FEI Tecnai 12 G2 instrument (120kV) and the Gatan slow-scan camera, using a low-dose regime to slow down the crystallization of vitrified water and to delay the formation of other artifacts due to beam damage. All images from both dry- and cryo-TEM (including electron diffraction patterns) were analyzed using Digital Micrograph Gatan Inc. software.

Differential Pulse Voltammetry (DPV). DPV was carried out using a CHI 760C potentiostat at 25 ± 2 °C in 0.2 M $LiClO_4$ electrolyte solutions, fitted with 2-mm glassy-carbon, Pt-wire and Ag/AgCl (3 M NaCl)[7] working, counter and reference electrodes respectively. Prior to data acquisition, solutions were purged with purified N_2 for 30 min. The following parameters were used: scan rate = 100 mV/s, sample width = 20 ms, pulse amplitude = 60 mV, pulse period = (interval) 200 ms, quiet time = 2 s, and sensitivity = 10^{-6} A/V. Routine cyclic voltammograms (CVs) were obtained under N_2 using the same cell and electrodes.

Preparation of [α-$PW_{11}O_{39}$Ti]–O^--complexed anatase-TiO_2 NCs (3) (Na^+ salt). This method is slightly modified improvement of the previously published protocol.[8] Titanium(IV) tetra-isopropoxide (TTIP, 100 μL) was diluted with 900 μL of isopropanol, and 236 μL of this solution (22.7 mg, 0.08 mmol TTIP) was added dropwise with vigorous stirring to 122 mg (0.04 mmol) of Na_7[α-$PW_{11}O_{39}$]·12H_2O in 9.76 mL water at ambient temperature. This gave 10 mL of a cloudy (milky white) pH-6.0 mixture, with final concentrations of 8 mM Ti(IV) and 4 mM Na_7[α-$PW_{11}O_{39}$]. Heating for 24 h at 170 °C in a 23 mL Teflon-lined 316 stainless-steel reaction vessel gave an optically clear, colorless pH-6.5 solution.

Preparation of [α-$SiW_{11}O_{39}$Ti]–O^--complexed anatase-TiO_2 NCs (2) (K^+ salt). This method is very similar to the previously published protocol.[8] Titanium(IV) tetra-isopropoxide (TTIP, 100 μL) was diluted with 900 μL of isopropanol, and 236 μL of this solution (68.2 mg, 0.08 mmol TTIP) was added dropwise with vigorous stirring to 129 mg (0.04 mmol) of K_8[α-$SiW_{11}O_{39}$]·13H_2O (**2**) in 9.76 mL water at ambient temperature. This gave 10 mL of a cloudy (milky white) pH-6.0 mixture, with a final concentrations of 8 mM Ti(IV) and 4 mM **1**. Heated for 24 h at 170 °C in a 45 mL Teflon-lined 316 stainless-steel reaction vessel gave an optically clear, colorless pH-6.7 solution.

Preparation of [α-$AlW_{11}O_{39}$Ti]–O^--complexed anatase-TiO_2 NCs (1) (K^+ salt). This method is very similar to that of complex **3**. Titanium(IV) tetra-isopropoxide (TTIP, 100 μL) was diluted with 900 μL of isopropanol, and 236 μL of this solution (68.2 mg, 0.08 mmol TTIP) was added dropwise with vigorous stirring to 130 mg (0.04 mmol) of K_9[α-$AlW_{11}O_{39}$]·13H_2O (**3**) in 9.76 mL water at ambient temperature. This gave 10 mL of a cloudy (milky white) pH-6.0 mixture, with a final concentrations of 8 mM Ti(IV) and 4 mM **1**. Heated for 24 h at 170 °C in a 45 mL Teflon-lined 316 stainless-steel reaction vessel gave a very slightly cloudy solution of pH-6.7.

Preparation of [$α_2$-$P_2W_{17}O_{61}$]–O^--complexed anatase-TiO_2 NCs (4) (K^+ salt). This method is very similar to the earlier method. Titanium(IV) tetra-isopropoxide (TTIP, 100 μL) was diluted with 900 μL of isopropanol, and 236 μL of this solution (68.2 mg, 0.08 mmol TTIP) was added dropwise with vigorous stirring to 225 mg (0.04 mmol) of K_{10}[$α_2$-$P_2W_{17}O_{61}$]·20H_2O (**4**) in 9.76 mL water at ambient temperature. This gave 10 mL of a cloudy (milky white) pH-6.0 mixture, with a final concentrations of 8 mM Ti(IV) and 4 mM **1**. Heated for 24 h at 170 °C in a 45 mL Teflon-lined 316 stainless-steel reaction vessel gave a very slightly cloudy solution of pH-6.7.

Isolation and purification of the POM-complexed anatase-TiO_2 NCs. The general protocol was as follows: The 10 mL reaction mixture was treated with 2 M NaCl, which immediately gives a cloudy solution. Under these conditions, reversible aggregation of the Ti-derivative of POM-capped anatase-TiO_2 nanocrystals (NCs) decreases their solubility in water, so they can be separated from the supernatant solution by centrifugation (30 min at 6000 rpm). This removes a significant amount of the polyoxometalate by-products, which consist primarily of α-$X^{n+}TiW_{11}O_{40}^{(10-n)-}$ (X= P^{5+}, Si^{4+}, Al^{3+}) and $α_2$-$P_2TiW_{17}O_{62}^{8-}$; Ti-derivative of the lacunary-POM reactants. After decanting the supernatant solution by pipette, the product, a hydrated white solid (ca. 10 mg), is collected and dissolved in 10 mL of pure water, to give a clear, colorless solution. Any POM anions remaining in the product are removed by first precipitating the nano-sized POM-complexed TiO_2 NCs by addition of NaCl (to a final concentration of 2 M). Two additional "washing" cycles of precipitation by addition of NaCl, followed by centrifugation and redissolution in pure water, are carried out to be certain that trace amounts of POM byproducts are no longer present. (After these "washing" cycles, the amount of "free" POMs present in the supernatant solution are already below the detection limit of UV-spectroscopy, i.e., less than 2 μM.). To remove excess NaCl used to isolate the complexed NCs, the thrice precipitated and redissolved solution was placed in a cellulose membrane

and dialyzed against pure water (1 L) in a 2 L beaker for 60 h, during which time, the water outside the dialysis membrane was replaced every 10-12 hours.

Characterization of the POM-complexed anatase-TiO_2 NCs. Characterization of the TiO_2 cores was easily accomplished using routine solid-state methods. However, the characterization of ligands on the surfaces of colloidal particles in solution—let alone their atomic-level connectivity to the particle surface—is extraordinarily challenging. This was achieved using a variety of solid- and solution-state microscopic, diffraction, and spectroscopic methods, combined with chemical digestion / etching, and electrochemistry. The use of these methods is described in ref. [8], and results for complexes, **1**, **2** and **4** are provided below.

Sample preparation for differential pulse voltammetry (DPV). Purified complexes, **1, 2, 3** and **4**, were dissolved in 200 mM of aq $LiClO_4$, to a final TiO_2-NC concentration of 1.7 μM.

Isolation and ESI-MS identification of Ti-substituted POMs after HCl digestion of the TiO_2 cores of POM-complexed anatase-TiO_2 NCs. To exhaustively cleave all the POM ligands in complexes **1**, **2**, **3** and **4** from the TiO_2 NCs, the TiO_2 cores were digested in conc. HCl. For this, a 20 mL volume of each complex (obtained from a single synthesis and extensively purified as described above), was treated with conc. HCl at 90 °C for 2 h. During this time, Ti(IV) atoms of the anatase NCs dissolved, giving a clear solution. Next, 1 mL of a 100 mM aq solution of tetrabutylammonium bromide (TBABr) was added to the digested-NC solution. After a few minutes, the solution became slightly cloudy. A white solid was collected by centrifugation, washed several times with pure water, and dissolved in MeCN. If the MeCN solution was slightly cloudy, it was clarified by filtration through a 20 nm Millipore filter. The clear MeCN solutions were analyzed by ESI-MS. Prior to analysis, a small amount of 10 mM aq NH_4Cl (to 1% by volume) was added to the MeCN solution to improve ionization.

Synthesis of titanium derivative of POMs α-$X^{n+}TiW_{11}O_{40}^{(10-n)-}$ (X= Al^{3+}, Si^{4+}, P^{5+}) and $α_2$-$P_2TiW_{17}O_{62}^{8-}$.

Synthesis of pure K_7[α-$AlTiW_{11}O_{40}$]. $K_9AlW_{11}O_{39}.13H_2O$ (10.4 g, 3.2 mmol) is taken in a round bottom flask. 10 mL deionized water was added to it. The pH of the solutions is around 6.6. Now, 2.508 mL $TiOSO_4$ (15 wt%) solution is added dropwise to it with continuous stirring. Immediately the pH of the solution decreased to 0.9. The stirring was continued for 30 min and then the solution was heated at 60 °C for 2 h. Then 4 g KCl was added to this solution and kept aside for few 2 days to obtain crude product as a white solid. The product was further recrystallized by another 2-3 times before use. Final yield of the product is ~ 29%. The final product was characterized with ^{27}Al NMR, FTIR, ESI-Mass spectra. IR (1200-600 cm^{-1}): 950, 880, 797, 756, 651 (weak); ^{27}Al NMR (D_2O): δ -71.8 ppm ($\nabla\nu_{1/2}$ = 22 Hz). The positive-ion ESI-MS spectrum (m/z 2000-4000) of the tetrabutylammonium salt of the POM in CH_3CN gave m/z 3952.57 as the most intense peak. This peak is attributed to $[(TBA)_5H_3AlTiW_{11}O_{40}]^+$ ion.

Synthesis of pure K_6[α-$SiTiW_{11}O_{40}$]. $K_8SiW_{11}O_{39}.15H_2O$ (10.2 g, 3.2 mmol) is taken in a round bottom flask. 10 mL deionized water was added to it. The pH of the solutions is around 6.6. Now, 2.508 mL $TiOSO_4$ (15 wt%) solution is added dropwise to it with continuous stirring. Immediately the pH of the solution decreased to 0.9. The stirring was continued for 30 min and then the solution was heated at 60 °C for 2 h. Then 5 g KCl was added to this solution and it was aside for few 2 days to obtain crude product as a white solid. The product was further recrystallized by another 2-3 times before use. Final yield of the product is ~ 33%. The final product was characterized with ^{29}Si NMR, FTIR, ESI-Mass spectra. IR (1200-600 cm^{-1}): 1011, 968, 913, 787, 654; ^{29}Si NMR (D_2O): δ -84.6 ppm. The positive-ion ESI-MS spectrum (m/z 2000-4000) of the tetrabutylammonium salt of the POM in CH_3CN gave m/z 3952.59 as the most intense peak. This peak is attributed to $[(TBA)_5H_2SiTiW_{11}O_{40}]^+$ ion.

Synthesis of pure Na_5[α-$PTiW_{11}O_{40}$] A slightly modified synthetic procedure was used for the synthesis of pure α-$PTiW_{11}O_{40}^{5-}$ as reported by Detusheva et, al.[9] 10.7 g (3.5 mmol) of $Na_7PW_{11}O_{39}.12H_2O$ is taken in a round bottom flask. 10 mL deionized water was added to it. The pH of the solutions is around 6.5. Now, 2.782 mL $TiOSO_4$ (15 wt%) solution is added dropwise to it with continuous stirring. Immediately the pH of the solution went to 0.75 and the solution becomes light yellow in color. The stirring was continued for 30 min and then the

solution was heated at 60 °C for 2 h. Then 5 g NaCl was added to this solution and kept aside for few days to get the product of $PTiW_{11}O_{40}^{5-}$. The product was further recrystallized by another 2-3 times before use. Final yield of the product is ~ 30%. The final product was characterized with ^{31}P NMR, FTIR, ESI-Mass spectra. IR (1200-600 cm^{-1}): 1073, 973, 894, 800, 660 (weak); ^{31}P NMR (D_2O): δ -13.9 ppm. The positive-ion ESI-MS spectrum (m/z 2000-4000) of the tetrabutylammonium salt of the POM in CH_3CN gave m/z 3954.59 as the most intense peak. This peak is attributed to $[(TBA)_5HPTiW_{11}O_{40}]^+$ ion.

Synthesis of pure α_2-$P_2TiW_{17}O_{62}^{8-}$. Pure α_2-$P_2TiW_{17}O_{62}^{8-}$ was synthesized according to the synthetic procedure reported by Yoshida et, al. [10] The final product was characterized with ^{31}P NMR, FTIR, ESI-Mass spectra. IR (1200-600 cm^{-1}): 1086, 1019, 950, 916, 785, 673 (weak), 600; ^{31}P NMR (D_2O): δ -10.4, -14.0 ppm. The positive-ion ESI-MS spectrum (m/z 2000-4000) of the K^+ salt of the POM in CH_3CN gave m/z 2308.69 (Z = 2) as the most intense peak. This peak is attributed to $[K_{10}P_2TiW_{17}O_{62}]^{2+}$ ion.

Photocatalytic production of H_2. Photocatalytic H_2 production was carried out in a three-mL gas-tight quartz cuvette connected to an upper glass bulb with a headspace volume of 16 mL. Carefully weighed samples of complexes **1**, **2**, **3** and **4**, of molecular clusters, α-$X^{n+}TiW_{11}O_{40}^{(10-n)-}$ (X= Al^{3+}, Si^{4+}, P^{5+}) and α_2-$P_2TiW_{17}O_{62}^{8-}$(Ti-derivative analogs of the Ti-substituted ligands) and commercial anatase were each solubilized/dispersed in 10% v:v methanol/water solutions. Each solution was purged with pure N_2 (g) for at least 30 min and then irradiated for 8 h using a 150 W Xe lamp (USHIO inc. Japan). H_2 in the headspace was quantified every two h by injecting 0.5 mL volumes into a gas chromatograph (Focus GC, Thermo Scientific) operating at isothermal conditions (40 °C) using a ShinCarbon ST micropacked column (0.53 mm diameter, 2 m length) equipped with a thermal conductivity detector and Ar as carrier gas. H_2 evolution rates reported in Figure 3, d and e of the text were calculated based on the masses of TiO_2, either cores of the complexes, or of pure commercial TiO_2. Values for the POM control were based on the mass of the POM itself (see Figure S14).

Photooxidation of MeOH and Redox Titrations: As mentioned above, the photocatalytic oxidation of MeOH was carried out in a three-mL gas-tight quartz cuvette connected to an upper glass bulb. Carefully weighed samples of (1, 2, 3, 4)-capped anatase-TiO_2 NCs, were each solubilized/dispersed in 10% v:v methanol/water solutions. Each solution was purged with pure N_2 (g) for at least 30 min and then irradiated for 8 h using a 150 W Xe lamp as mentioned above. After photoirradiation, the number of photoexcited e^-s present in reduced (1, 2, 3, 4)-capped anatase-TiO_2 NCs were determined by the redox titration using $Na_2Cr_2O_7$ (5mM) as the oxidant. All four highly reduced TiO_2 complexes were reacted gradually (~ 10ul in each time) with the $Na_2Cr_2O_7$ oxidant under Inert conditions and the decrease in the intensity of the excited e^-s (blue coloration) were monitored by UV-vis absorption spectroscopy. For the method, see: Schrauben et al.; Science 336, 1298 (2012).

Calculations of proton uptake: See Figures S22-S23 and related discussion.

Quantum yield measurements: The quantum yields (QYs) of the molecular of Ti(IV)-substituted POMs, α-$X^{n+}TiW_{11}O_{40}^{(10-n)-}$ (X= Al^{3+}, Si^{4+}, P^{5+}) and α_2-$P_2TiW_{17}O_{62}^{8-}$ was carried out using the methods reported by Hatchard et al.[11] The photon flux of the light source was calculated using chemical actinometer techniques. Potassium ferrioxalate was used as a standard for the chemical actinometer techniques. The average intensity of the light is measured to be 1.8×10^{-9} einstein/s. The quantum yields of the POMs were calculated at 280 nm. All 4 titanium derivatives of the POMs were irradiated for 2 h using UV-Vis irradiations in the presence of 10% v:v methanol/water solutions. The amount of photoreduction for each POM was calculated from their respective molar extinction coefficients for the one electron reduction conditions. The QYs for the one electron reduction of the POMs, 0.0303, 0.0354, 0.0572 and 0.044 for the POMs listed above, respectively, are plotted in Figure 4 of the text. The published uncertainty in the ferrioxalate actinometer method of Hatchard and Parker[11] is ±2% under ideal conditions. When combined with an estimated maximum uncertainty of ±3% in the extinction coefficients of the adsorption maxima of the one-electron reduced Ti(IV)-substituted POMs, the total uncertainty increases to ca. ±5%. This value was used to generate the error bars shown in Figure 4 of the text.

Estimation of the number of POM ligands on each NC. Based on the particle size and W to Ti ratio, it is possible to estimate the number POM ligands on each average-sized NC. This is done by fitting calculated to experimental W to Ti ratios with use of the surface area of an average-sized NC. The surface coverage by bound ligands is then used as a variable with more or less surface area allocated to each ligand until the calculated W to Ti ratio matches that determined by EDX. In ongoing work and publications on this class of nanostructures we find that the surface coverage by bound POM ligands varies considerably depending on the type of POM ligand and nature of the complexed metal-oxide, metal-hydroxide or metal-oxyhydroxide NC core. Surface coverage is varied by allocating specific areas or "footprints" to the POM ligands. In the present work, these surface-area allocations varied from 1.78 to 2.5 nm^2, which is in line with results obtained for other POM-complexed NCs. Based on this, we find between 86 to 90 POM ligands for the series of four complexes reported here. Importantly, these values refer to the numbers of ligands on the average-sized TiO_2 cores determined by statistical analyses of histograms of particle sizes observed in TEM images. Smaller or larger than average NC cores will obviously be ligated by smaller or larger numbers of ligands. Another source of uncertainty arises from the non-homogeneous shapes of the NCs. For this reason, we assign a conservative uncertainty of ± 15 ligands to the values determined for each complex. This is source of the value reported in the abstract and text, of 90 ± 15 POM ligands per complex. This value of ± 15 ligands is the result of sensitivity analysis of the effect of variation in particle shape on calculated W to Ti ratios for specific POM footprint values. The fitting parameters and results are provided in Table 1 of the text.

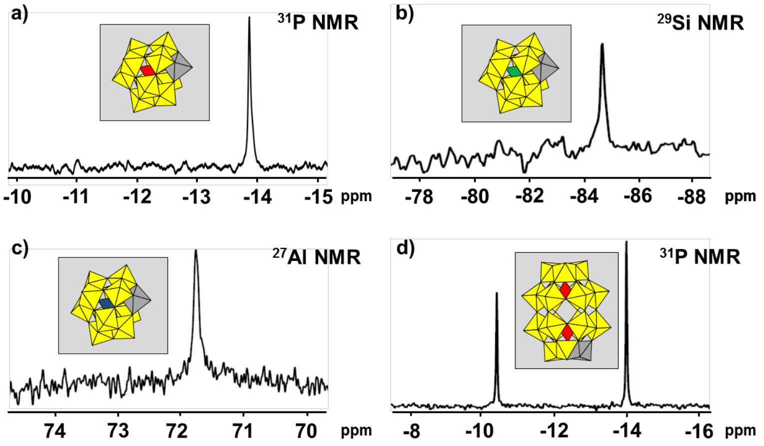

Figure S1. NMR spectra obtained after reactions of amorphous TiO_2(s) with α-$X^{n+}W_{11}O_{39}^{(12-n)-}$ (X= Al^{3+}, Si^{4+}, P^{5+}) and α$_2$-$P_2W_{17}O_{61}^{10-}$ for 24 h at 170 °C (see Experimental Methods). After heating, the lacunary POMs are consumed to form the corresponding Ti-derivatives, a fraction of which remain coordinated to the TiO_2 NCs. Approx. one half of the lacunary POM-anion reactant is hydrolyzed to phosphate. (The corresponding signals are not included in the NMR spectra but see Ref.[8] for details and discussion). **a**) P-31 NMR spectrum of the reaction mixture revealing the formation of [α-$PTiW_{11}O_{40}$]$^{5-}$ by product (-13.9 ppm). **b**) Si-29 NMR spectrum of the reaction mixture revealing the formation of [α-$SiTiW_{11}O_{40}$]$^{6-}$ (-84.6 ppm). **c**) Al-27 NMR spectra of the reaction mixture revealing the formation of [α-$AlTiW_{11}O_{40}$]$^{7-}$ (-71.8 ppm). **d**) P-31 NMR spectra of the reaction mixture revealing the formation of [α$_2$-$P_2TiW_{17}O_{62}$]$^{8-}$ (-10.4 and -14.0 ppm).

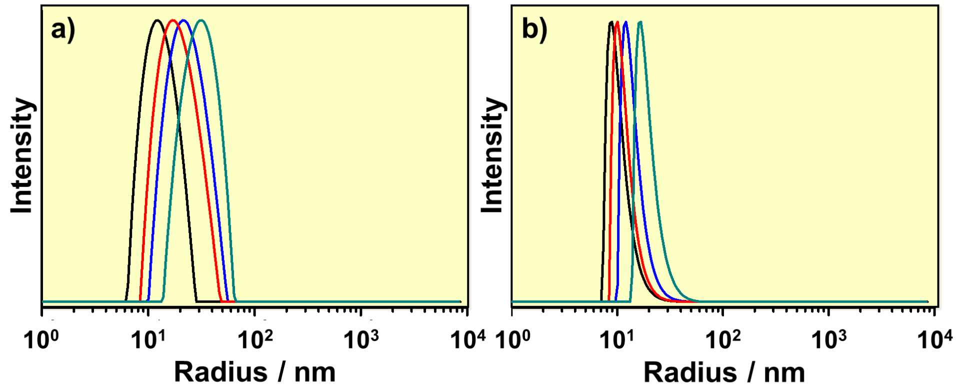

Figure S2. Dynamic light scattering (DLS) data for aqueous solutions obtained after syntheses of POM-complexed TiO_2 NCs (see Experimental Methods). Panel **a)** shows the unweighted intensity profile of the four solutions (CONTIN method), while panel **b)** shows the number-weighted intensities. In both panels, the **black** curve indicates the DLS data obtained immediately after the reaction of amorphous TiO_2(s) with Na_7[α-$PW_{11}O_{39}$] at 170 °C, the **red**, **blue** and **cyan** curves show the DLS results obtained after reactions with of amorphous TiO_2(s) with K_8[α-$SiW_{11}O_{39}$], K_9[α-$AlW_{11}O_{39}$] and K_{10}[α$_2$-$P_2W_{17}O_{67}$], respectively. In all cases, optically clear solutions were observed, with no precipitation or very little cloudiness (filtered prior to DLS analysis). In the unweighted intensity plots (panel **a**), the average hydrodynamic radii increased from ca. 12 (**black** curve) to 18 (**red** curve) to 22 nm (**blue** curve) to 30 nm (**cyan** curve) as the formal charges of the Ti-substituted POM ligands (including the full charges of the bridging oxo linkages) increased incrementally, from 5- to 6- to 7- to 8- respectively, resulting in greater degrees of electrostatic aggregation. The average number-weighted radii, panel **b)**, similarly increased from ca. 9 (**black** curve) to 11 (**red** curve) to 14 nm (**blue** curve) to18 nm (**cyan** curve).

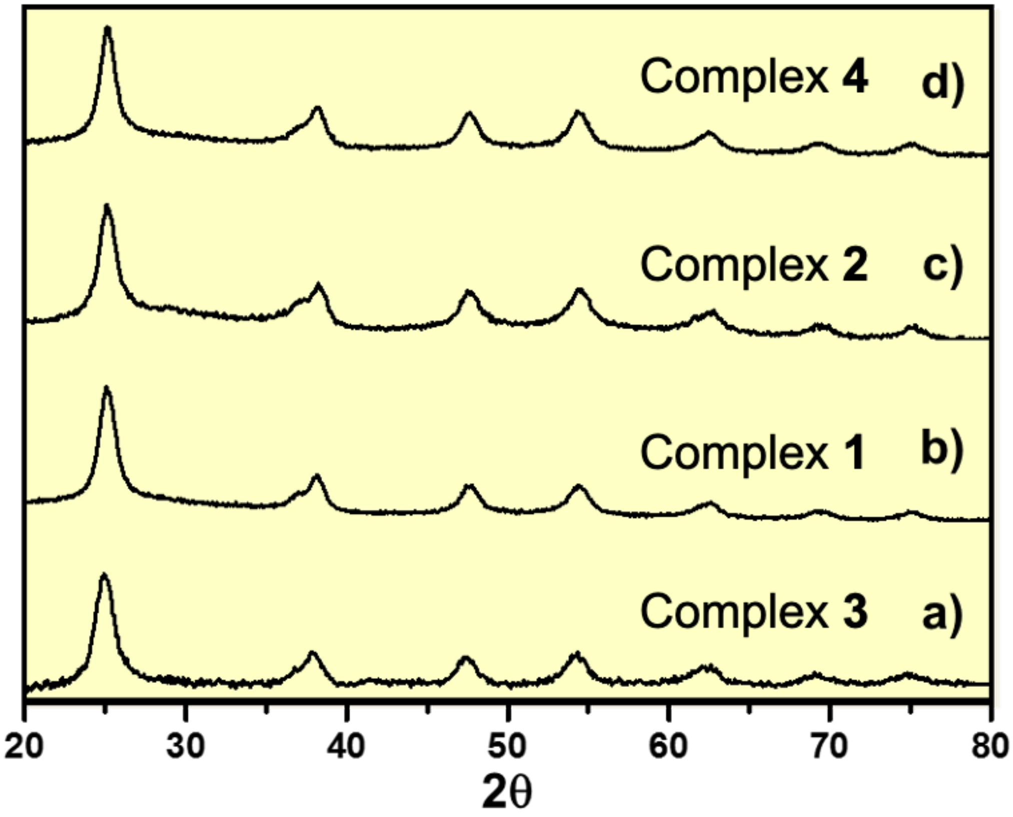

Figure S3. Powder X-ray diffraction of the anatase-TiO_2 nanocrystal cores of the four complexes after purification by repeated precipitation / redissolution, followed by dialysis (see experimental section for details). The PXRD patterns presented here are: **a**) complex **3**; **b**) complex **1**; **c**) complex **2**; **d**) complex **4**. The size of the anatase-TiO_2 crystallites was determined using the Debye-Scherrer's equation, $L = 0.9\lambda/\beta \mathrm{Cos}\Theta$, where, L is the size of the crystallites, λ is the X-ray wavelength (nm), β is the peak width at half maximum (in radians) of the most intense diffraction peak, and θ is the Bragg angle of diffraction. Calculated values are 6.1 ± 0.2 nm, 6.5 ± 0.2 nm, 6.2 ± 0.2 nm and 6.8 ± 0.3 nm for complexes **3**, **1**, **2**, and **4**, respectively.

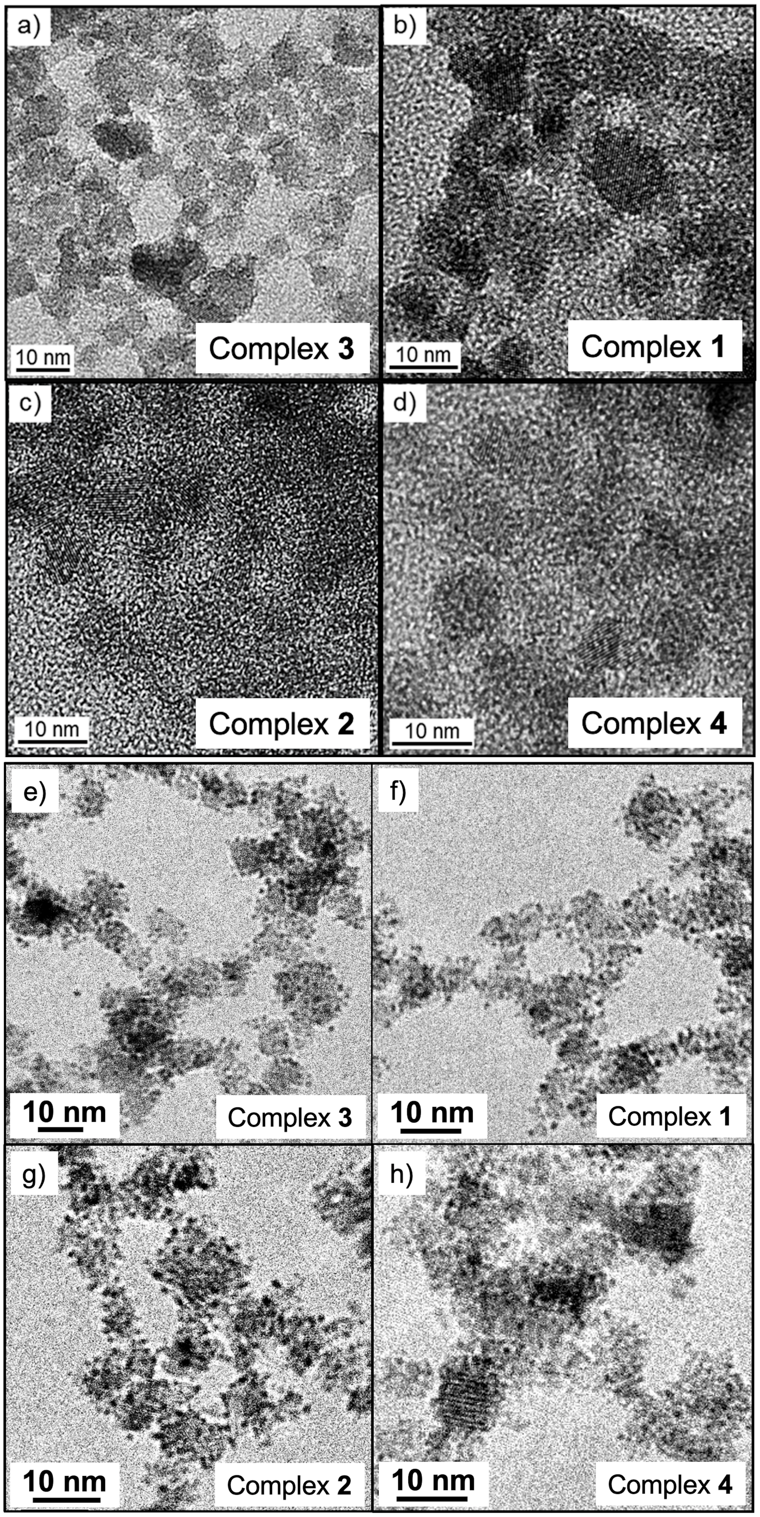

Figure S4. Bright-field and cryo-TEM images of the four complexes after purification by repeated precipitation / redissolution, followed by dialysis (see experimental section for details). Bright-field images are: **a**) complex **3**; **b**) complex **1**; **c**) complex **2**; **d**) complex **4**. Panels **e**), **f**), **g**) and **h**) show cryo-TEM images of the four complexes, **3**, **1**, **2** and **4**, respectively. Due to phase-contrast imaging, the cryo-TEM images reveal the POM ligands at the particle surfaces. The particle association observed in these images is due to labile electrostatic interactions involving the counter-cation inherently associated with the POM ligands.

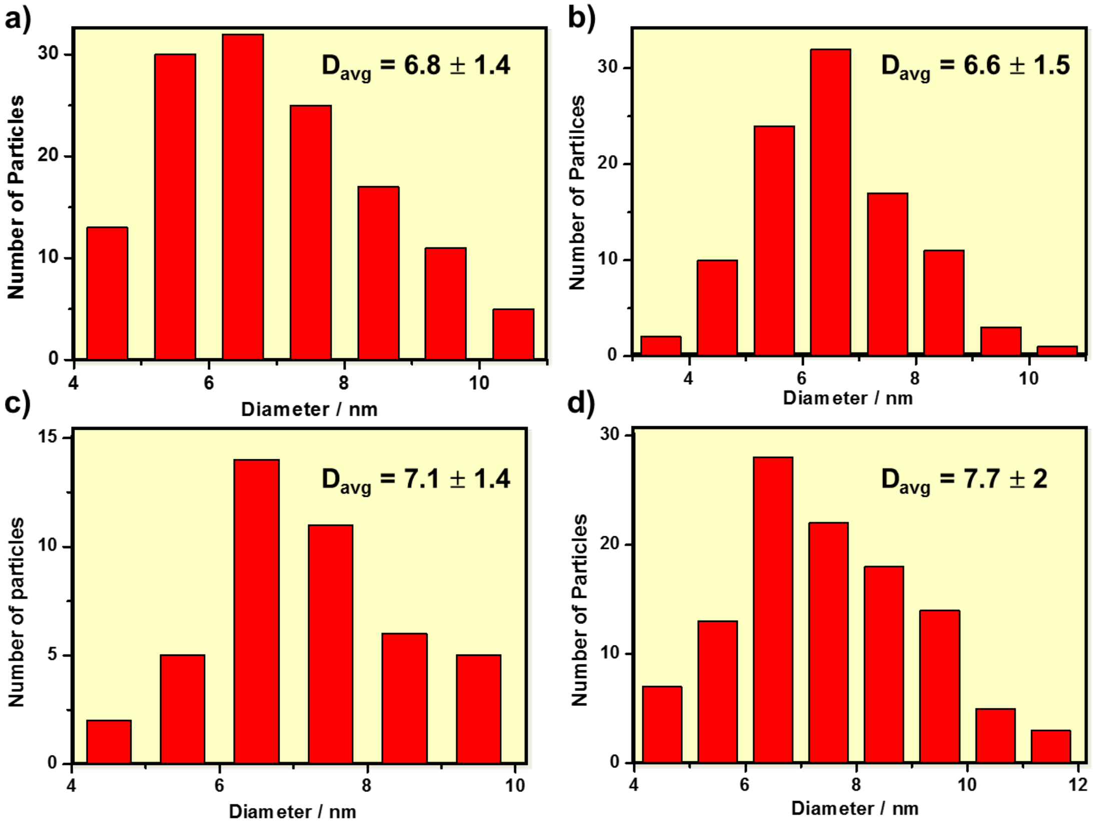

Figure S5. The histograms of the particle size in diameter and the average particle sizes of POM-TiO_2 complexes after purification, calculated from bright field TEM images, examples of which are shown above in Figure S4. The histograms presented here are for: (**a**) Complex **3**, (**b**) complex **2**, (**c**) complex **1**, and (**d**) complex **4**. The average particle size obtained are 6.8 ± 1.4 nm, 7.1 ± 1.4 nm, 6.6 ± 1.5 nm, and 7.7 ± 2 nm for complexes **3**, **2**, **1**, and **4**, respectively.

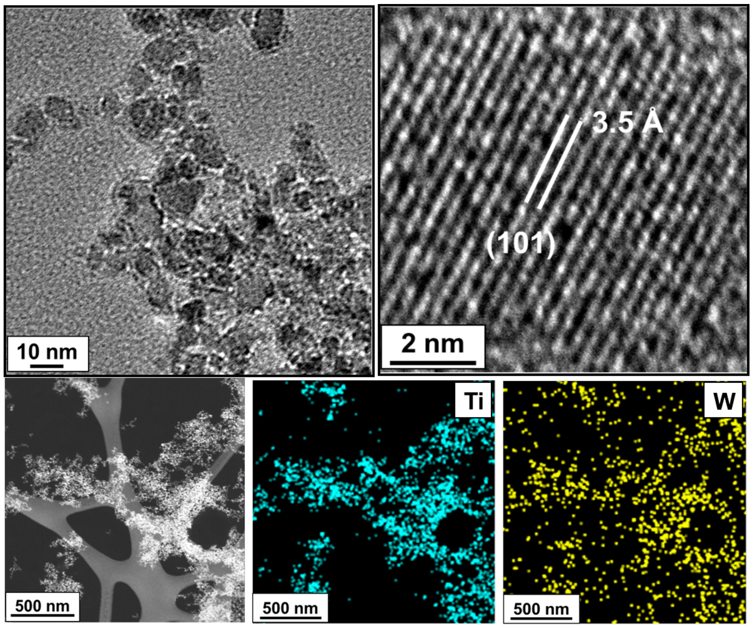

Map Sum Spectrum	Atomic %
**Ti**	86.84
**W**	13.16
**Total**	100.00

Length (TEM histogram)	width	height
7.1	7.1	4.0
Volume of TiO2 NC	201.64	nm^3
Density of TiO2	3.89E-21	g nm^-3
MW of TiO2	79.87	g mol-1
# Ti atoms per particle	5.9150E+03	
Surface area of TiO2 NC	214.42	nm^2
POM footprint	2.5	nm^2
# POM per NC	86	
Total number of W+Ti ato	6.8585E+03	

	**Calculated**	**Experimental (from EDX)**
Atom/atom fraction of Ti=	0.862	86.8
Atom/atom fraction of W=	0.138	13.2

Figure S6. Bright field and HRTEM images of the anatase-TiO_2 nanocrystal cores ligated by $[AlTiW_{11}O_{40}]^{7-}$ (complex **1**). The lower panels of the figures provide dark field images along with the elemental mapping for Ti and W atoms. **Central table**: The atomic weight percents of Ti and W are 87% and 13% (excluding other elements, such as, O and Al). **Bottom table**: Fitting of calculated Ti and W atom fractions to experimental values by varying the number of POM ligands per average sized NC core obtained from the histograms of TEM images (Figure S5).

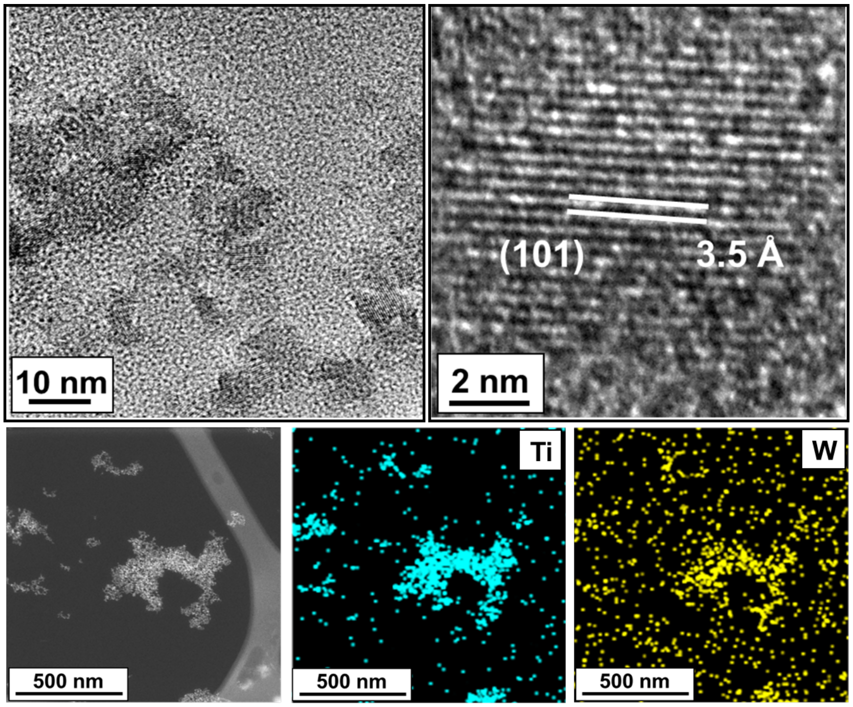

Map Sum Spectrum	Atomic %
**Ti**	80.36
**W**	19.64
**Total**	100.00

Length (TEM histogram)	width	height
6.6	4.5	4.5
Volume of TiO2 NC	133.65	nm^3
Density of TiO2	3.89E-21	g nm^-3
MW of TiO2	79.87	g mol-1
# Ti atoms per particle	3.9206E+03	
Surface area of TiO2 NC	154.8	nm^2
POM footprint	1.8	nm^2
# POM per NC	86	
Total number of W+Ti atoms	4.8666E+03	
	**Calculated**	**Experimental (from EDX)**
Atom/atom fraction of Ti=	0.806	0.804
Atom/atom fraction of W=	0.194	0.196

***Figure S7*.** Bright field and HRTEM images of the anatase-TiO_2 nanocrystal cores ligated by $[SiTiW_{11}O_{40}]^{6-}$ (complex **2**). The lower panels of the figures provide dark field images along with the elemental mapping for Ti and W atoms. **Central table**: The atomic weight percents of Ti and W are 80% and 20% (excluding other elements, such as, O and Si). **Bottom table**: Fitting of calculated Ti and W atom fractions to experimental values by varying the number of POM ligands per average sized NC core obtained from the histograms of TEM images (Figure S5).

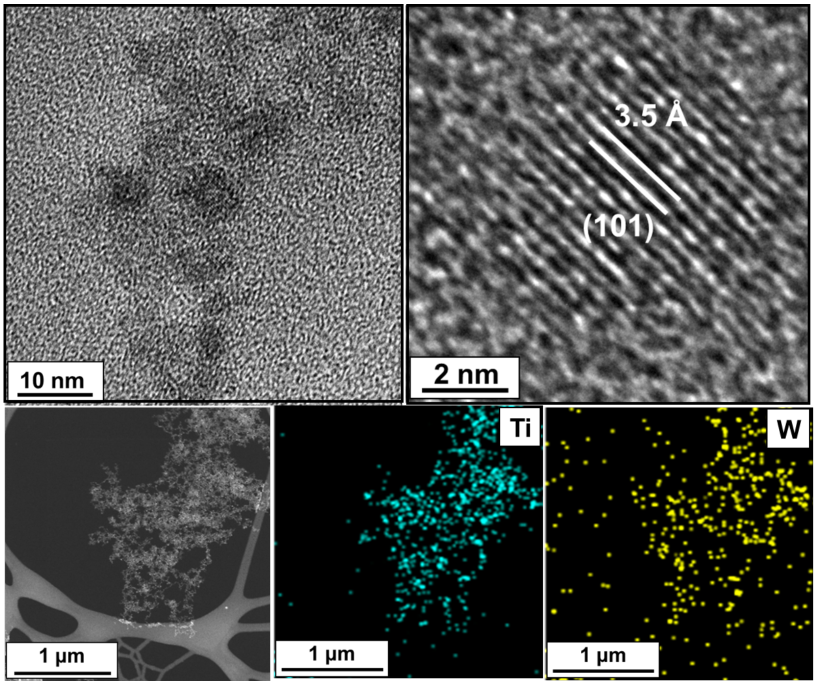

Map Sum Spectrum	Atomic %
**Ti**	77
**W**	23
**Total**	100.00

Length (TEM histogram)	width	height
7.7	4.7	4.7
Volume of TiO2 NC	170.09	nm^3
Density of TiO2	3.89E-21	g nm^-3
MW of TiO2	79.87	g mol-1
# Ti atoms per particle	4.9896E+03	
Surface area of TiO2 NC	188.94	nm^2
POM footprint	2.1	nm^2
# POM per NC	90	
Total number of W+Ti atoms	6.5191E+03	
	**Calculated**	**Experimental (from EDX)**
Atom/atom fraction of Ti=	0.765	0.77
Atom/atom fraction of W=	0.235	0.23

Figure S8. Bright field and HRTEM images of the anatase-TiO_2 nanocrystal cores ligated by $[P_2TiW_{171}O_{62}]^{8-}$ (complex **4**). The lower panels of the figures provide dark field images along with the elemental mapping for Ti and W atoms. **Central table**: The atomic weight percents of Ti and W are 77% and 23% (excluding other elements, such as, O and P). **Bottom table**: Fitting of calculated Ti and W atom fractions to experimental values by varying the number of POM ligands per average sized NC core obtained from the histograms of TEM images (Figure S5).

Note: Previously-reported data[8] for complex **3**, from the same techniques used in Figures S6-S8 gave atomic weight percents of Ti and W equal to 75% and 25%. More accutate data, recently obtained by ESI-MS give 80% Ti and 20% W. The latter values were used in the present work. The updated fitting data are presented here:

Length (TEM histogram)	width	height
6.8	4.5	4.5
Volume of TiO2 NC	137.70	nm^3
Density of TiO2	3.89E-21	g nm^-3
MW of TiO2	79.87	g mol-1
# Ti atoms per particle	4.0394E+03	
Surface area of TiO2 NC	162.9	nm^2
POM footprint	1.81	nm^2
# POM per NC	90	
Total number of W+Ti atoms	5.0294E+03	

	**Calculated**	**Experimental (from EDX)**
Atom/atom fraction of Ti=	0.803	0.804
Atom/atom fraction of W=	0.197	0.196

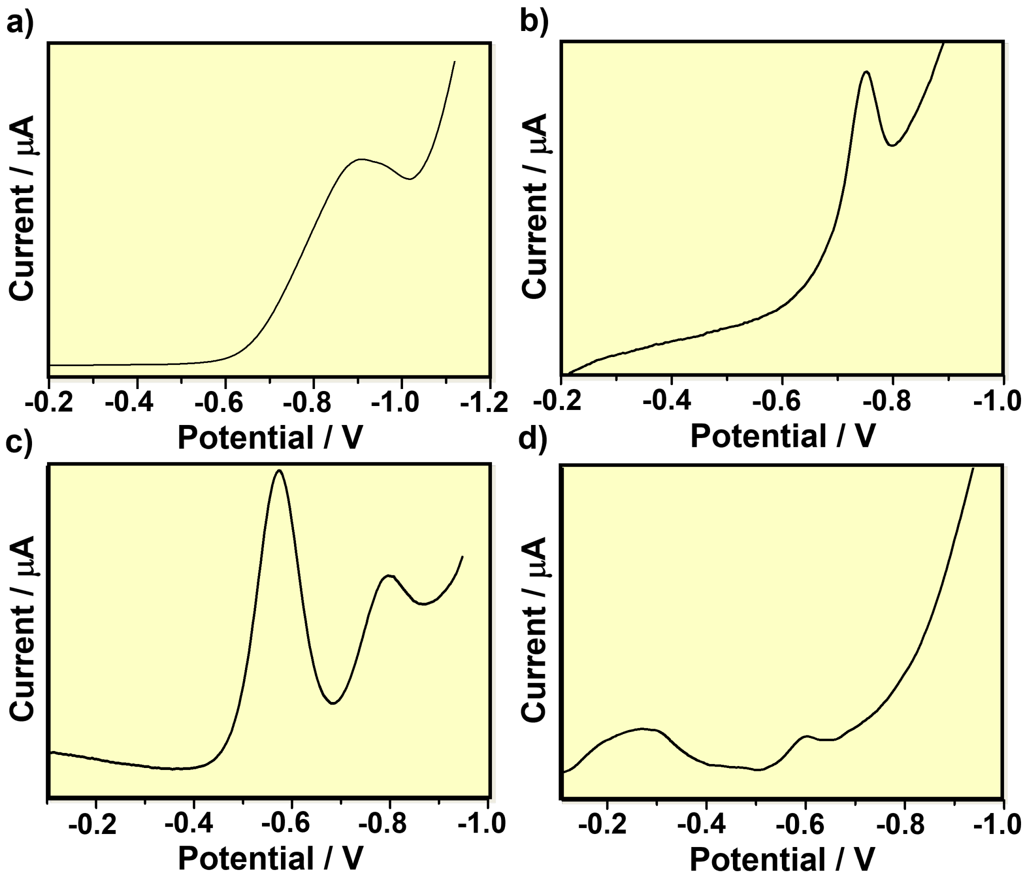

Figure S9. Forward differential pulse voltammograms (DPVs) showing first one-electron reductions of POM anions when ligated to TiO_2 (in 0.2 M aqueous $LiClO_4$; vs. Ag/AgCl, 2M NaCl) in: (**a**) complex **1**, (**b**) complex **2**, (**c**) complex **3** and (**d**) complex **4**. The respective potentials (as shown in each panel) are: -910mV, -750mV, 575 mV.[8] and 280mV. These values are reported relative to the NHE in Figure 3a of the text.

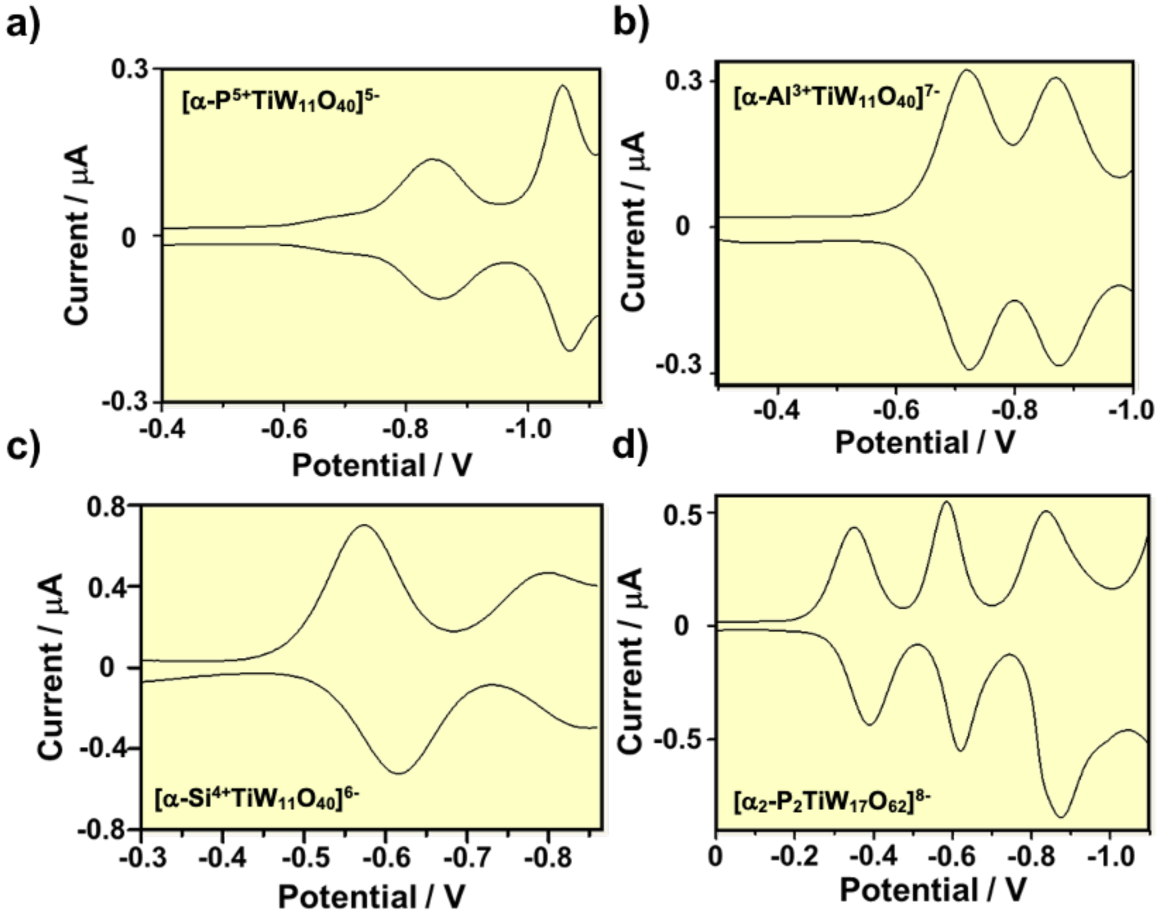

Figure S10. Cyclic voltammograms of molecular Ti^{IV}=O substituted POM anions, $[\alpha\text{-}X^{n+}Ti(O)W_{11}O_{39}]^{(10-n)-}$ (X^{n+} = Al^{3+}, Si^{4+}, P^{5+}) and $[\alpha_2\text{-}P_2Ti(O)W_{17}O_{61}]^{8-}$, deployed as models for the POM ligands in complexes **1**-**4** **(**0.2 M aqueous $LiClO_4$, vs. Ag/AgCl 2M NaCl). The molecular POMs analyzed here are: **a**) $[\alpha\text{-}P^{5+}TiW_{11}O_{40}]^{5-}$ (related to the ligands in complex **3**), **b**) $[\alpha\text{-}Al^{3+}TiW_{11}O_{40}]^{6-}$ (related to the ligands in complex **1**), **c**) $[\alpha\text{-}Si^{4+}TiW_{11}O_{40}]^{6-}$ (related to the ligands in complex **2**), **d**) $[\alpha_2\text{-}P_2TiW_{17}O_{62}]^{8-}$(related to the ligands in complex **4**). The 1st one-electron reduction potentials of the molecular POMs are (from **a** to **d**) -575mV, -720mV, -844mV and -350mV vs. Ag/AgCl (2M NaCl). These potentials are shown in Figure 3b of the text, relative to the NHE.

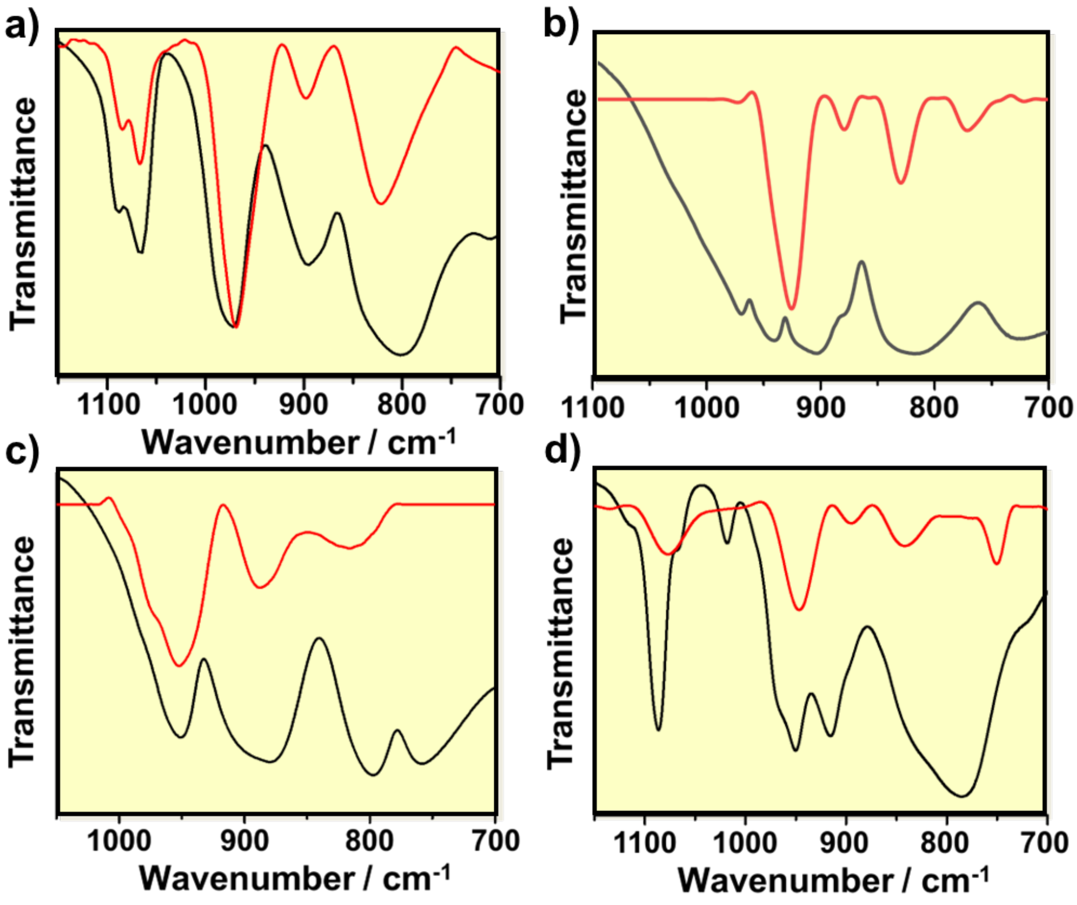

Figure S11. The FTIR spectra of Ti-substituted POMs and the baseline corrected spectra of related POM-complexed TiO_2 NCs. **a**) The unprocessed FTIR spectrum of pure $Na_5[\alpha\text{-}PTiW_{11}O_{40}]$ (black curve) and the baseline corrected (red curve) FTIR spectrum of the related complex **3** (KBr pellet). **b**) The unprocessed FTIR spectrum of pure $K_6[\alpha\text{-}SiTiW_{11}O_{40}]$ (black curve) and the baseline corrected (red curve) FTIR spectrum of the related complex **2** (KBr pellet). **c**) The unprocessed FTIR spectrum of pure $K_7[\alpha\text{-}AlTiW_{11}O_{40}]$ (black curve) and the baseline corrected (red curve) FTIR spectrum of complex **1** (KBr pellet). **d**) The unprocessed FTIR spectrum of pure $K_8[\alpha\text{-}P_2TiW_{17}O_{62}]$ (black curve) and the baseline corrected (red curve) FTIR spectrum of the related complex **4** (KBr pellet). Acquisition of these spectra was non-trivial, due to the minor presence of the POM ligands on the surfaces of the NCs. Nevertheless, in all but one case (panel **b**) the final spectra, even after baseline correction, which artificially over- or under-emphasized specific peak intensities, were in line with expectations. Definitive characterization of the bound ligands of all complexes (including that shown in panel **b**) was secured by ICP-MS after acid-digestion of the TiO_2 cores and protolytic cleavage of the bound ligands: see **Figures S12** and **S13**.

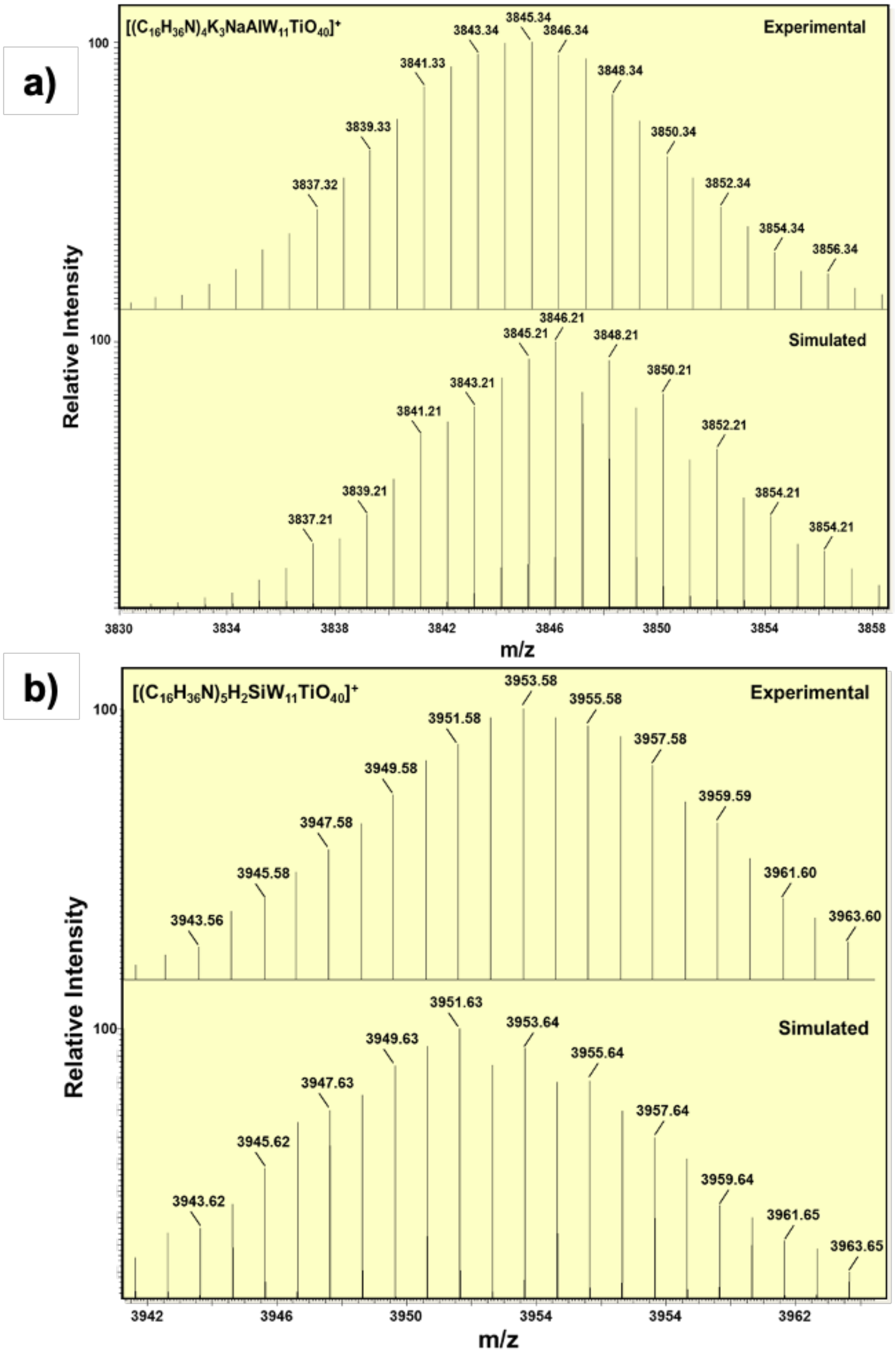

Figure S12. ESI mass spectra obtained after digesting **a**) complex **1**, and **b**) complex **2**, using conc. HCl, isolating the *n*-Bu_4N salts of the POMs as white solids, and dissolving the solids in acetonitrile (see experimental section above for details). The spectrum shows one 1+ ions which can be simulated precisely Corresponding envelopes of simulated signals are provided below each experimentally obtained spectrum.

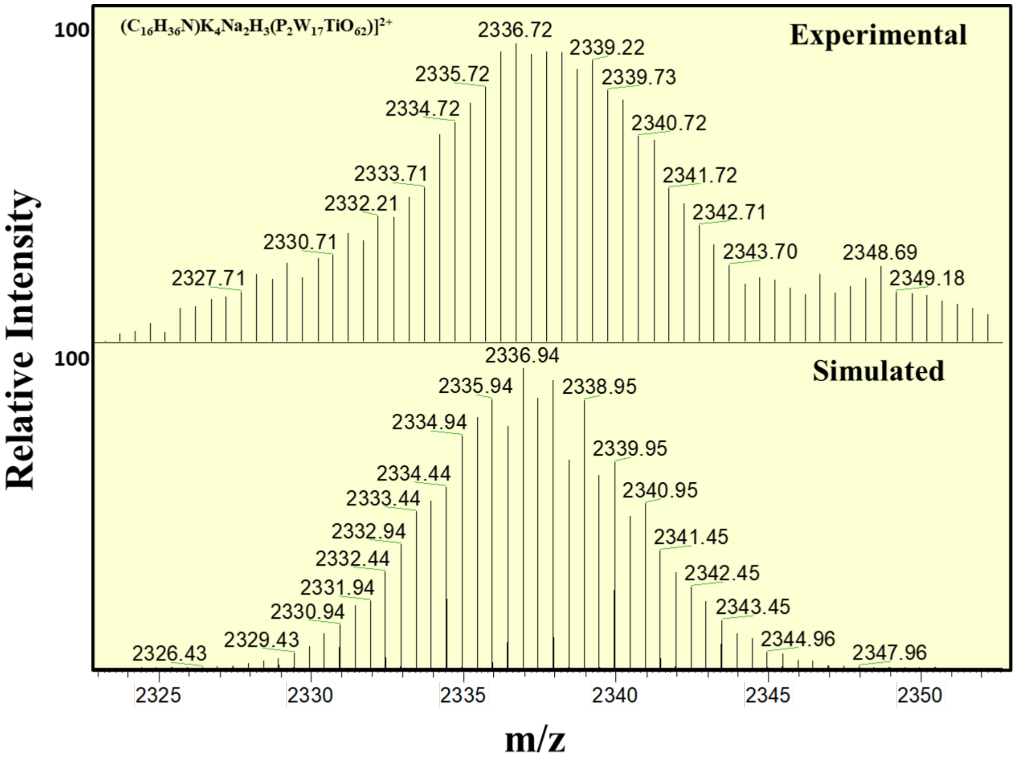

Figure S13. ESI mass spectra obtained after digesting complex **4**, using conc. HCl, isolating its *n*-Bu_4N salt as a white solid, and dissolving the solid in acetonitrile (see experimental section above for details). The spectrum shows one 2+ ions which can be simulated precisely; simulated signals are provided below the experimental spectrum.

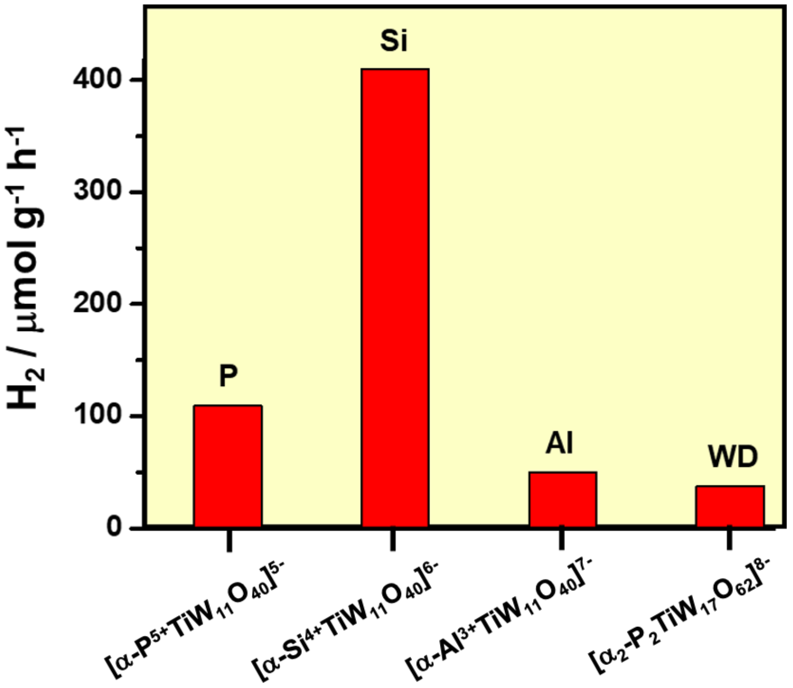

Figure S14. Rates of H_2 evolution for the titanium-substituted POMs deployed as analogs of the Ti-substituted POMs ligated to complexes **1** to **4**. The reaction conditions, including near-neutral pH values were the same as those used in H_2 evolution by the POM-complexed TiO_2 NCs themselves. Notably, the most active POM cluster-anion for photochemical H_2 evolution under those conditions is [□-$SiTiW_{11}O_{40}$]$^{6-}$, closely related to the oxo-donor ligands in complex **2**. However, the most active POM-complexed TiO_2 (complex **3**) is ligated by [□-$PW_{11}O_{39}Ti$]-O^- anions. As such, the data provided here show that the large rate of H_2 evolution observed for complex **3** is not due to H_2 evolution by the POM-ligand alone.

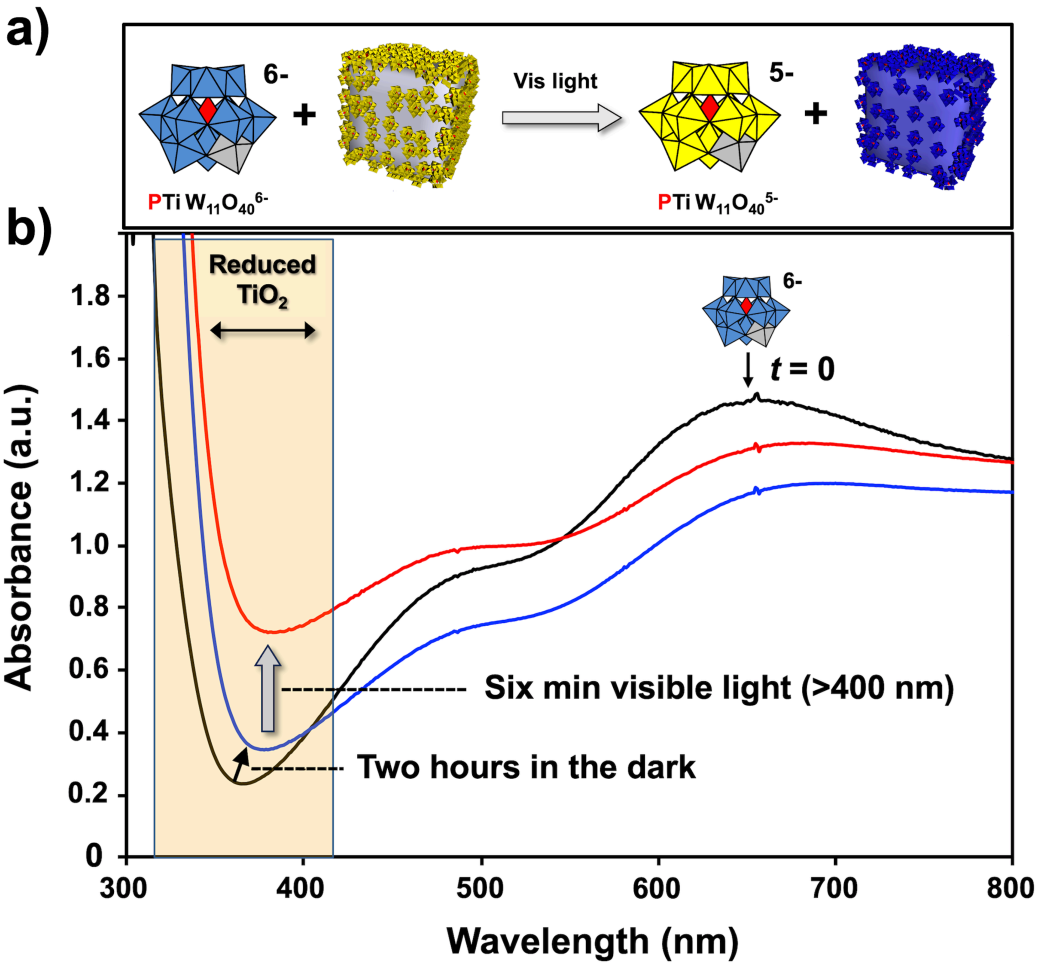

Figure S15. Indirect evidence of visible-light driven electron injection from reduced POM ligands into complexed TiO_2. The key to this reaction was the observation that at 385 nm, the absorbance arising from reduced Ti-substituted Keggin anions is small, while reduced TiO_2 has significant absorbance at this wavelength. This was used to monitor the reaction shown in panel **a**). This reaction was carried out by first reducing molecular anions, α-$[P^{5+}TiW_{11}O_{40}]^{5-}$ (analogs for the ligands in complex **3**) in water (10% v/v MeOH) under UV-Vis irradiation. This gave the reduced POM anions (black curve in panel **b**), in which its two absorbance maxima, at ca. 475 and 645 nm, are clearly observed. Note that the absorbance of the reduced POM at 360-390 nm is relatively small. Then, in the dark, fully oxidized complex **3**, i.e., fully oxidized TiO_2 ligated by oxidized $[\alpha\text{-}PW_{11}O_{39}Ti]\text{-}O^-$ donor ligands, was added. After two hours in the dark, an increase in absorbance at 385 nm indicated a small degree of TiO_2 reduction (blue curve in panel **b**). The spectrum remained unchanged for an additional hour in the dark. At the that time, the sample was irradiated by visible light (150 W Xe lamp equipped with a 400-nm cutoff filter). In only six minutes, a significant increase in absorbance for reduced TiO_2 was observed, as indicated by a jump in absorbance from the blue to red curves in panel **b**, highlighted by the grey arrow. This jump in absorbance at 385 nm after only 6 min of visible-light irradiation shows that electrons from the reduced POM in solution are rapidly injected into the TiO_2 core of **3** by visible light. Based on the very similar reduction potentials of the POM ligand on TiO_2 in complex **3**, and reduction potential of the Ti-substituted analog for this ligand (see Figures 2a and 2b of the text) it is reasonable to expect a significant degree of electron transfer from the reduced POM in solution to the ligands on TiO_2. As such, the visible light would drive electron injection from the reduced ligand into TiO_2. At the end of the day, however, this assumption is difficult to prove experimentally due to the similar potentials at which the ligands on TiO_2, and TiO_2 itself, are reduced. (Efforts to selectively reduce the ligands electrochemically were not successful.) Because this step was found to be central to the overall mechanism of H_2 evolution, DFT calculations were carried out, and confirmed that visible light does indeed drive electron injection from reduced POM ligands into the ligated TiO_2 core (see Figure 4 of the text, and the data immediately below in this SI file).

UV-vis spectrum simulation. To simulate the absorption spectrum of TiO_2 nanoparticles modified with $Na_5PW_{11}TiO_{40}$ and reduced $Na_6PW_{11}TiO_{40}$ ($Na_6PW^VW^{VI}_{10}$ TiO_{40}), we considered two finite models with POMs adsorption on a $(TiO_2)_{38}$ cluster, which is constructed by appropriately "cutting" an anatase slab exposing the majority (101) surface. The charge of the POM was compensated by using the equivalent sodium atoms displayed around the POM. Geometry optimizations for the clusters are performed using the projector augment wave (PAW) method implemented in the Vienna ab initio simulation package (VASP).[12-13] The electron exchange and correlation potential are treated with the Perdew-Wang 91 functional (PW91) and the kinetic energy cut-off was set to 500 eV.[14] [13] The DFT+U[15] approach is used to describe the TiO_2, by setting U = 6.0 eV and J = 0.5 eV to treat the 3d electrons of Ti atoms.[16] The reciprocal space for the clusters was described with only Γ point. All optimization reaches until self-consistence with thresholds of 1×10^{-5} eV and $-3\cdot10^{-2}$ $eV\cdot A^{-1}$ for the electronic and the ionic convergence, respectively. The optimized models are displayed in Figure S16. The optimized geometries are then utilized to the single point time dependent density functional theory (TD-DFT) studies for the lowest 80 transitions, by using B3LYP functional together with 3-21G* and LANL2DZ/6-31G* basis sets for TiO_2 and POM respectively in Gaussian 09.[17-18] The water effect was considered with the CPCM model.[19] Finally, with the aid of Multiwfn 3.2 software, the UV-vis spectrum for the designed model was generated as a sum of Gaussian curves with a 0.3 eV half-height width, as well as the transition analysis.[20]

Figure S17 presents the simulated UV-visible absorption spectra of pristine and composite systems calculated at the B3LYP level. For the isolated $(Ti_2O_2)_{38}$ cluster, a HOMO-LUMO (H-L) gap of 4.24 eV is observed, with the lowest TDDFT transition energy at 3.50 eV. This computed excitation energy aligns well with the experimental bandgap of TiO_2 nanoparticles (3.2 eV) and reported values for nanoscale TiO_2. The isolated $Na_5PW_{11}TiO_{40}$ species exhibits a slightly larger H-L gap of 4.35 eV, with its strongest oscillator strength transitions occurring at 4.42 eV. In the composite $Na_5PW_{11}TiO_{40}$-$(Ti_2O_2)_{38}$ system, the absorption band spans 3.1–3.7 eV (331–397 nm), peaking at 3.65 eV. Compared to pure TiO_2 (299–354 nm), the absorption edge of $Na_5PW_{11}TiO_{40}$-$(Ti_2O_2)_{38}$ shows a slight red shift. Key transitions contributing to the highest oscillator strengths (S59 and S75) are summarized in Table S1. The 340 nm transition predominantly involves a HOMO-3 → LUMO+5 excitation (32% contribution), characterized by oxygen ligand-to-Ti charge transfer within $(Ti_2O_2)_{38}$, as illustrated in Figure S18. Notably, this transition does not facilitate charge transfer to the polyoxometalate (POM). Further analysis of the lower-intensity peak at 3.12 eV (397 nm, state 6) reveals a dominant HOMO-5 → LUMO transition (77% contribution). Here, occupied orbitals are localized on TiO_2, while unoccupied states reside on the POM, suggesting potential visible-light-driven electron transfer from TiO_2 to the POM. This mechanism is corroborated by the electron density difference map (EDDM) in Figure S19, which visualizes electron redistribution from W orbitals to TiO_2 upon excitation.

On the other hand, the one electron reduced $Na_6PW_{11}TiO_{40}$ and the composite $Na_6PW_{11}TiO_{40}$-$(Ti_2O_2)_{38}$ both exhibit broad visible-region absorption bands. The $Na_6PW_{11}TiO_{40}$-$(Ti_2O_2)_{38}$ shows a pronounced red shift relative to TiO_2, extending absorption to 485 nm and effectively enhancing TiO_2's weak visible-light response. Simulated spectra reveal two prominent visible-region peaks at ~600 nm and ~1000 nm (Figure S17c), originating from the HOMO excitations localized on W(d) orbitals of the POM. The 622 nm transition (S0 → S38, 1.99 eV) arises from α-HOMO to α-LUMO+28 ((30, 31, and 34) excitations, involving Ti(3d) orbitals with minor W(4d) and O(2p) contributions, indicative of a metal-to-metal charge transfer (MMCT) mechanism from W(d) (POM) to Ti(3d) (TiO_2). The electron density difference map (EDDM) in Figure S19 directly visualizes electron redistribution from W(d) orbitals to TiO_2 under visible-light irradiation for S38. This interfacial coupling between POM and TiO_2 states likely promotes efficient electron injection. In contrast, the most important (α-LUMO+15 and α-LUMO+8) unoccupied orbitals for the adsorption at 913 (1.36 eV) and 1006 nm (1.23eV) are orbitals with significant W(d) contributions of POM, reflecting intramolecular excitations. Collectively, these DFT simulations elucidate how reduced POMs enhance TiO_2's visible-light absorption and validate the electron transfer pathways between the two components.

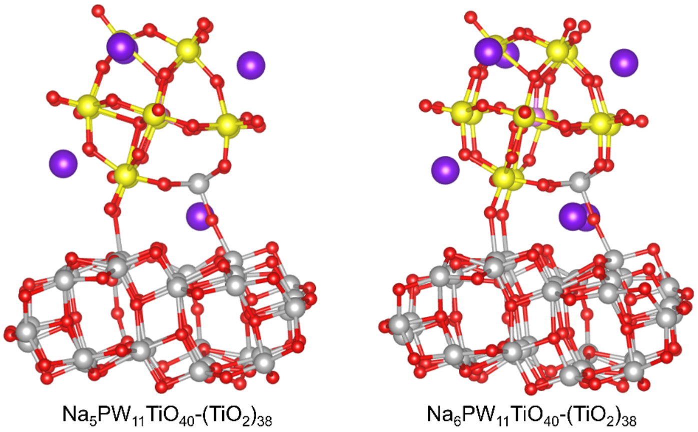

Figure S16. The optimized structure of $Na_5PW_{11}TiO_{40}$ and $Na_6PW_{11}TiO_{40}$ adsorbed on $(TiO_2)_{38}$ cluster.

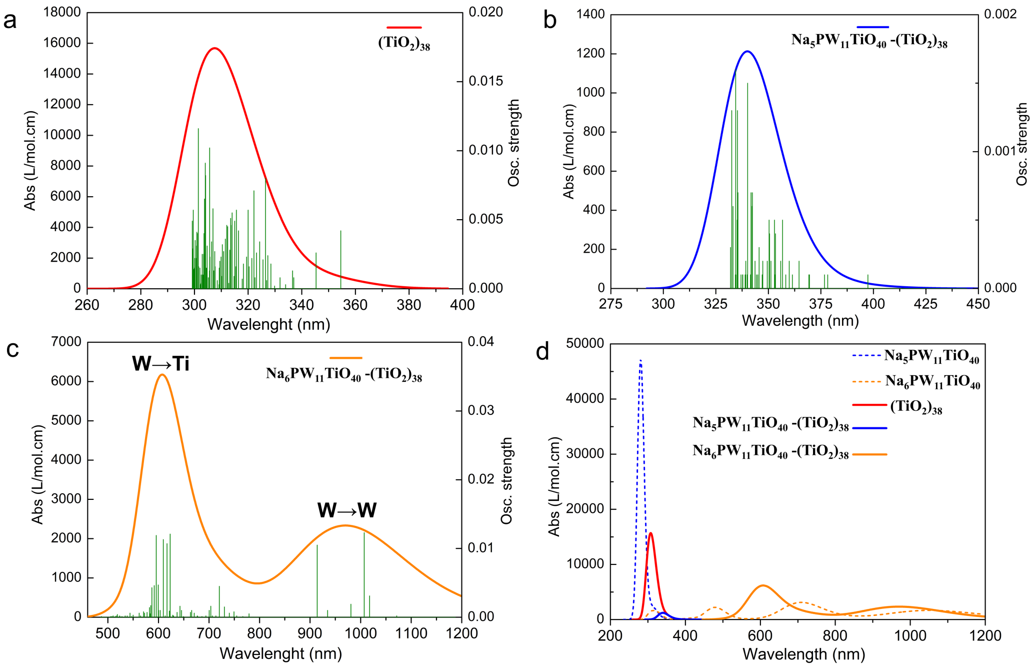

Figure S17. Comparison of the simulated UV-visible spectrum at B3LYP level for pure $(TiO_2)_{38}$ cluster (a), $(TiO_2)_{38}$-$Na_5PW_{11}TiO_{40}$ (b), $(TiO_2)_{38}$-$Na_6PW_{11}TiO_{40}$ (c), and all species (d).

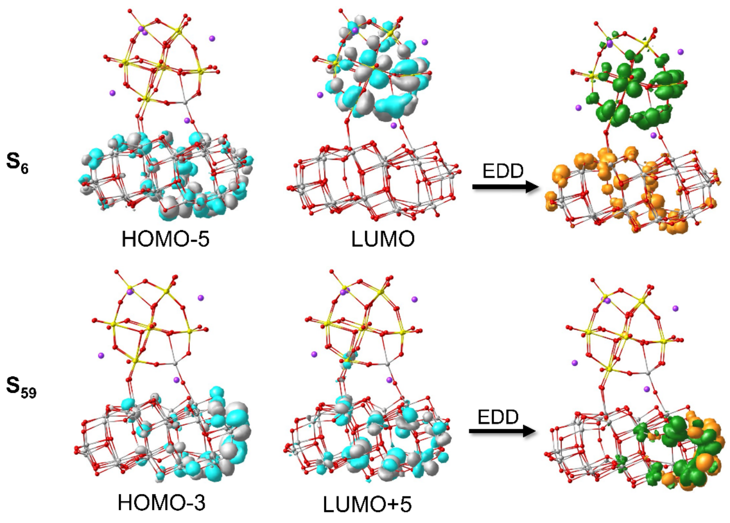

Figure S18. Molecular orbitals (isovalue = 0.02) and electron density difference (isovalue = 0.001, green indicates increasing of electron density, and orange means decreasing of electron density) representations involved in the S_6 and S_{59} transitions for the $(TiO_2)_{38}$-$Na_5PW_{11}TiO_{40}$. Electron density difference (EDD) shows that the HOMO-5 to LUMO transition involves electron transfer from titania to the POM. In the EDD representation green and yellow identify regions where the electron density increase and decrease, respectively.

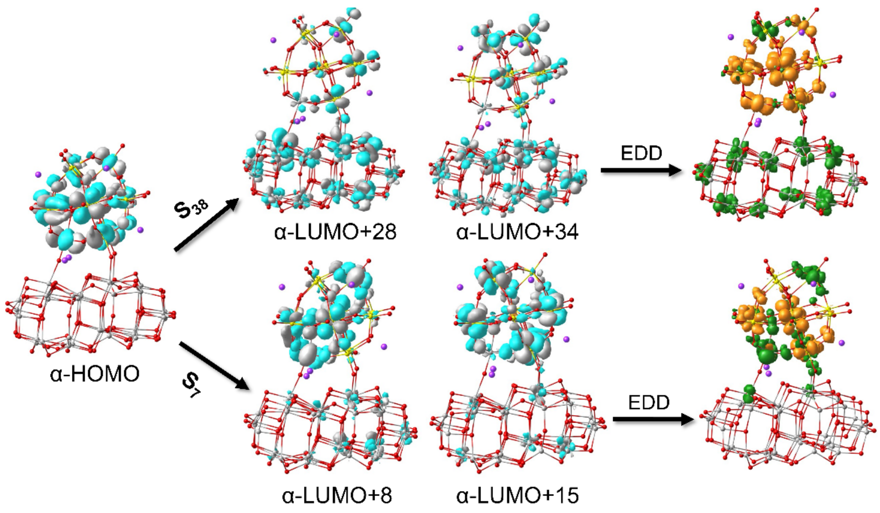

Figure S19. Molecular orbitals and electron density difference representations involved in the S_{38} and S_7 transitions for $(TiO_2)_{38}$-$Na_6PW_{11}TiO_{40}$. In the EDD representation green and yellow identify regions where the electron density increase and decrease, respectively.

Table S1. Excitation energy (E, eV), wavelength (λ, nm), oscillator strength (*f*) and composition of some representative transitions (S_n) in terms of MOs for $(TiO_2)_{38}$-$Na_5PW_{11}TiO_{40}$ and α-MOs for $(TiO_2)_{38}$-$Na_6PW_{11}TiO_{40}$.

S_n	λ_{max} (nm)	ΔE(eV)	*f*	Main contribution
**$Na_5PW_{11}TiO_{40}$ - $(TiO_2)_{38}$**				
**S0 → S59**	340	3.65	0.015	H-3→ L+5 (32%), H-3→ L+6 (12%)<br>H-3→ L+7 (9%), H-2→ L+6 (9%)
**S0 → S75**	334	3.71	0.016	H-8→ L+5 (8%), H-6→ L+5 (3%)<br>H-4→ L+5 (3%), H-3→ L+6 (3%)
**$Na_6PW_{11}TiO_{40}$ - $(TiO_2)_{38}$**				
**S0 → S46**	595	2.08	0.01	H → L+38 (13%), H → L+37(9%)<br>H → L+63 (11%), H → L+65 (9%)
**S0 → S42**	609	2.04	0.01	H → L+34 (10%), H → L+36 (8%)<br>H → L+62 (19%), H → L+65 (6%)
**S0 → S40**	616	2.01	0.01	H → L+30 (12%), H → L+31 (13%)<br>H → L+60 (20%), H → L+64 (11%)
**S0 → S38**	622	1.99	0.01	H → L+28 (14%), H → L+30 (11%)<br>H → L+31 (12%), H → L+34 (16%)
**S0 → S10**	913	1.36	0.01	H → L+15 (85%), H → L+18 (5%)
**S0 → S7**	1006	1.23	0.01	H → L+8 (69%), H → L+7 (7%)<br>H → L+9 (7%)

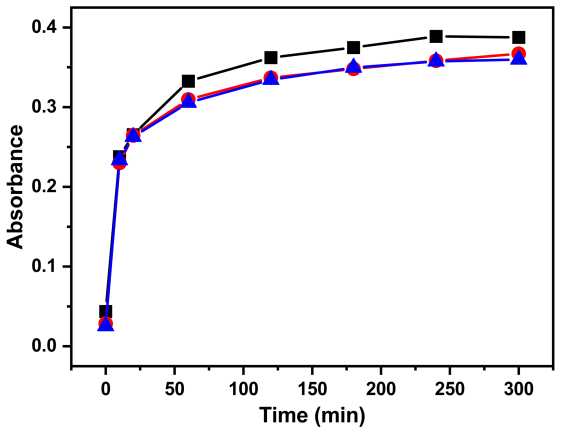

Figure S20. Absorbance vs. time data for the reduction of TiO_2 complexes of $[AlTiW_{11}O_{40}]^{7-}$(**1**; red circles), $[SiTiW_{11}O_{40}]^{6-}$ (**2**, blue triangles), and $[P_2TiW_{17}O_{62}]^{8-}$(**4**; Black Squares).

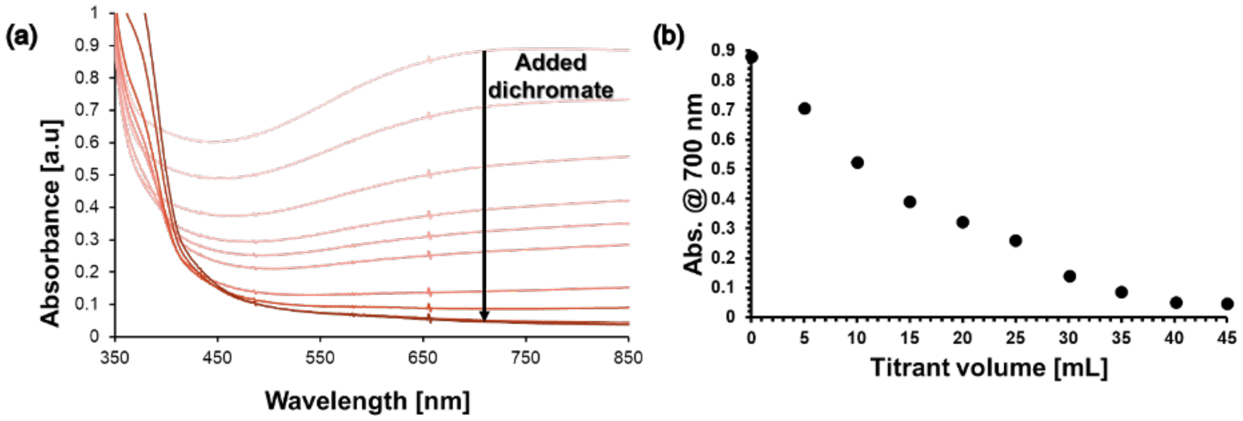

Figure S21. Visible-absorbance spectra (350-850 nm) obtained during the redox titration of complex, **3**, reduced by 8h of UV-Vis photoirradiation in the presence of 10% MeOH. Each curve shows the absorbance spectra after the incrmental additions of $Na_2Cr_2O_7$ (5 mM stock) solution. The absorbance of the complex decreased with each addition of dichromate. At the smaller wavelengths, it can be seen that when approaching the end of the titration, there is an excess of dichromate, and its absorbance starts to increase. This absorbance of excess dichromate was used later in calculating numbers of electrons in reduced **3**. **(b)** Titration curve for absorbance at 700 nm vs. volume of added dichromate.

Number of protons in reduced TiO_2 cores of 3. As previously noted, each NC was found to contain approximately 440 ± 40 electrons. This significant 440- negative charge within such a small crystal, with an average diameter of 6 nm, must be balanced. Because no alkali metals or any other plausible charge-balancing metal cations were present in solution, it was assumed that protons serve as charge balancers, originating from oxidation of MeOH used in the reduction process. To validate this assumption, the pH of the complex solution was measured before and after re-oxidation to quantify the number of protons released during oxidation.

For this, a cuvette containing a solution of reduced complex **3** was prepared by 8 h irradiation in 10% MeOH:water. The pH of the oxidized solution (before irradiation) was 7.01. Following reduction, the pH was effectively identical at 7.40. The reduced complex was then oxidized by redox titration using $AgNO_3$ under inert atmosphere, which resulted in the formation of silver nanoparticles and a color change from blue to orange-yellow (Figure S22), which was monitored by UV-Vis spectroscopy. When the absorbance indicated complete oxidation of the complex, the pH was 4.49. Based on calculations, if the proton-to-electron ratio in the reduced TiO_2 was 1:1, the expected pH after oxidation should have been 3.51, which is significantly lower than the experimentally observed value.

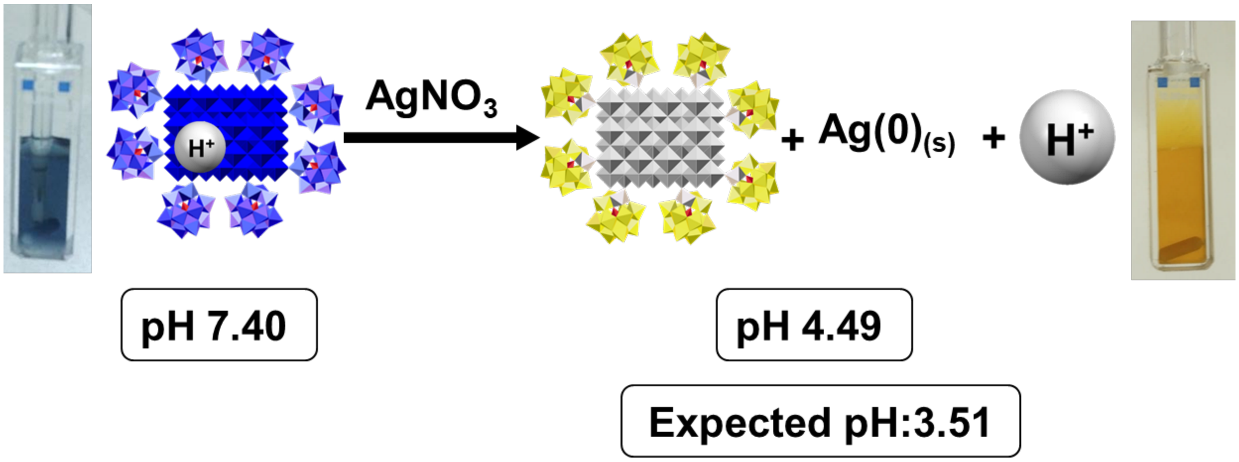

Figure S22. Titration of reduced complex **3** with silver nitrate ($AgNO_3$). The reduced complex solution is blue and has a pH of 7.40. When reacted with silver nitrate, the complex is oxidized, silver NPs are formed, and the solution's color turns to yellow orange. The pH at the end of the titration was 4.49, higher than the expected pH of 3.51 based on assumption of 1:1 ratio of electrons to protons.

This discrepancy was tentatively attributed to buffering by species generated during the photochemical oxidation of MeOH. To investigate this possibility, an experiment was conducted in which pH values were tracked as acid was quantitatively added in small increments to a reduced solution of the complex under an inert atmosphere (solid-black circles in Figure S23). The pH-titration curve that would have been observed in the absence of buffering was calculated, giving the open circles in Figure S23. These data demonstrated that species generated during photooxidation of MeOH indeed led to buffering behavior when protons were added.

Multiple attempts were made to identify the source of buffering in the reduced solution. It was hypothesized that a buffer consisting of formate and formic acid was generated from the oxidation of methanol in the solution. Efforts to identify and quantify these species were not successful. However, the empirical results were deemed analytically reliable.

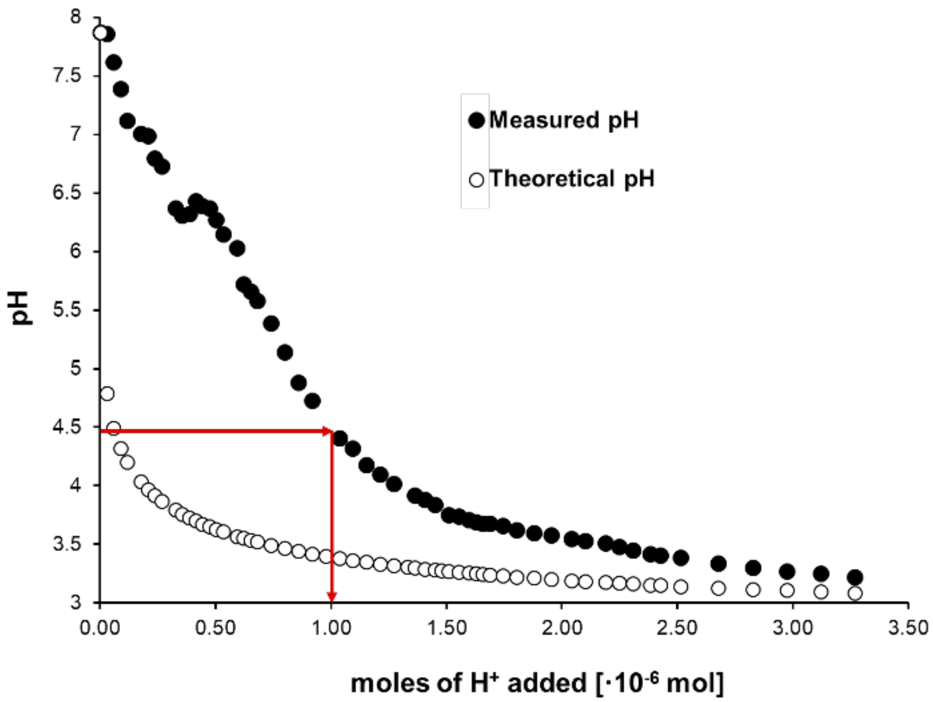

Figure S23. Acid addition to a reduced solution of complex **3**. **Black circles**: measured pH values as a function of added acid. **White circles**: theoretical pH values calculated based on the amount of acid added. A significant discrepancy is observed between the two curves, with the 'measured pH' curve (black circles) showing buffering behavior.

The buffer curve in Figure S23 (solid-black circles) was used as an analytical tool to determine the number of protons associated with the pH value of 4.49 obtained from redox titration (Figure S22). At a pH of 4.49 (the final pH in the titration with $AgNO_3$) the quantity of H^+ added to the solution was one micromole, as indicated by the red arrows in Figure S23. This finding led to the conclusion that upon re-oxidation of the complex during the reaction with $AgNO_3$, there was a release of 1 µmol of protons from the complex, which closely matched the 0.96 µmol of Ag^+ used to titrate the electrons in reduced complex **3** (Figure S22). This established a 1 to 0.96 ratio of protons to electrons in reduced **3**, i.e., 1.04 protons per electron, well within 10% uncertainty in the average number of electrons per NC.

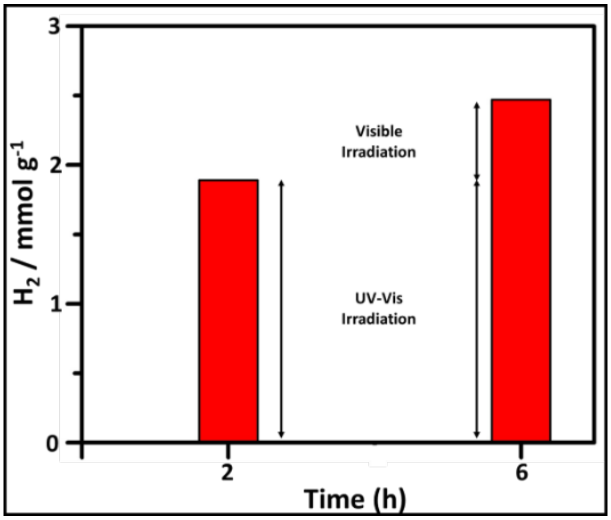

Figure S24. The role of visible light on hydrogen production was evaluated by the following experiment in which H_2 are measured using 10% MeOH-water mixture under two different experimental conditions. In first case, H_2 per g of [α-$PW_{11}O_{39}$Ti]–O–-complexed TiO_2 NCs (complex **3**) was irradiated with UV-Vis light. Subsequently, the same solution was further irradiated with only visible light for an additional 4h. The amount of H_2 produced in both cases are shown in the above figure. Once a steady state of TiO_2 reduction (300 e^- and 300 H^+ per NC) was reached after 2 h of UV-Vis irradiation, a 395 nm cut-off filter was used to permit irradiation by visible light alone. Notably, an additional 0.65 mmol H_2 (per g TiO_2) was produced under visible light alone. Due to "shut off" of the UV-driven photooxidation of MeOH by the POM ligands, this amount of H_2 was the stoichiometric product produced without additional electron / proton injection into TiO_2.

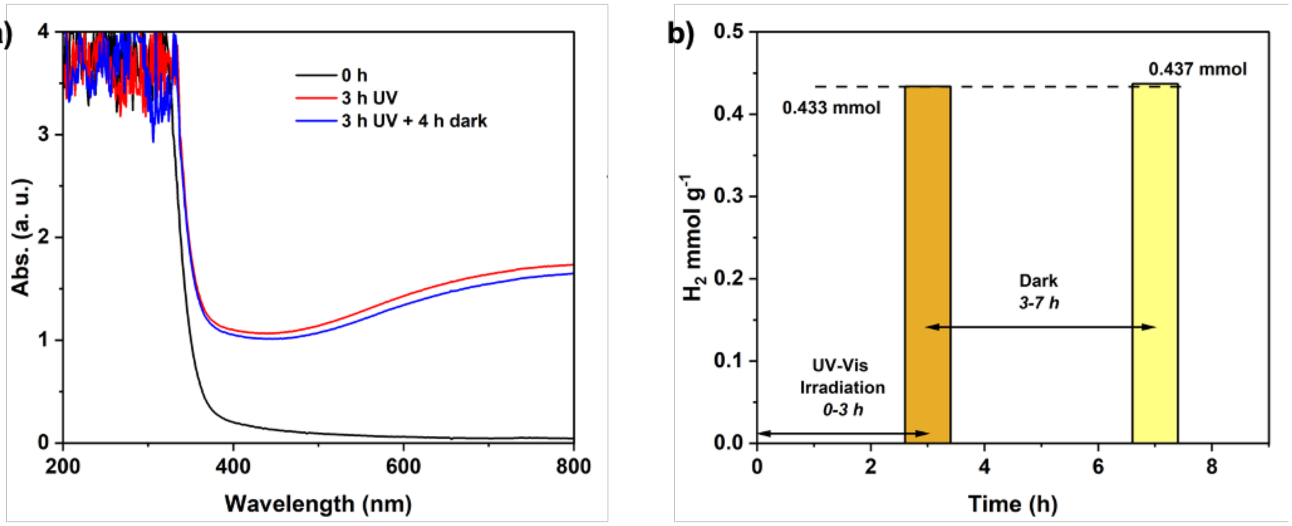

Figure S25. Control experiment showing that the H_2 evolution in Figure S24 is indeed driven by visible light, and does not occur spontaneously in the dark. A solution of [α-$PW_{11}O_{39}$Ti]–O–-complexed TiO_2 NCs (complex **3**) was subjected to UV-Vis photoirradiation in presence of 10% MeOH for 3 h. The reduced material gave rise to the UV-Vis spectrum (red curve) in panel **a**, while producing the amount of H_2 shown at the 3-h bar in panel **b**). Then, after 4 h in the dark, there was effectively no change in absorbance in the UV-Vis spectrum of the solution (panel **a**, blue curve) and no additional H_2 (to within experimental uncertainty) was produced, as indicated by the height of the 5-h bar in panel **b**.

References

[1] N. Haraguchi, Y. Okaue, T. Isobe, Y. Matsuda, *Inorg. Chem.* **1994**, *33*, 1015-1020.

[2] C. Tourné, G. Tourné, *Bull. Soc. Chim. Fr.* **1969**, *4*, 1124-1136.

[3] G. Maksimov, R. Maksimovskaya, O. Kholdeeva, M. Fedotov, V. Zaikovskii, V. Vasil'ev, S. Arzumanov, *J. Struct. Chem.* **2009**, *50*, 618-627.

[4] A. Tézé, G. Hervé, R. G. Finke, D. K. Lyon, in *Inorganic Syntheses, Vol. 27* (Ed.: A. P. Ginsberg), John Wiley & Sons, Inc., USA, **1990**, pp. 85.

[5] I. A. Weinstock, J. J. Cowan, E. M. G. Barbuzzi, H. Zeng, C. L. Hill, *J. Am. Chem. Soc.* **1999**, *121*, 4608-4617.

[6] Contant, R.; Klemperer, W. G.; Yaghi, O. In Inorganic Syntheses; Ginsberg, A. P., Ed.; John Wiley & Sons, Inc., USA: 1990; Vol. 27, p 104

[7] Y. V. Geletii, C. L. Hill, A. J. Bailey, K. I. Hardcastle, R. H. Atalla, I. A. Weinstock, *Inorg. Chem.* **2005**, *44*, 8955-8966.

[8] R., M., G. Gan-Or, M. Saganovich, O. Zeiri, Y. Wang, M. R. Chierotti, R. Gobetto, I. A. Weinstock, Angew. Chem. Int. Ed. 2015, 54, 12416-12421.

[9] L. G. Detusheva, M. A. Fedotav, L. L. Kuznetsova, A. A. Vlasov, G. A. Likholobov, *Russ. Chem. Bull.* **1997**, *46*, 874-880.

[10] S. Yoshida, H. Murakami, Y. Sakai, K. Nomiya, *Dalton Trans*. 2008, 4630-4638.

[11] C. G. Hatchard, C. A. Parker, *Proc. R. Soc. London, Ser. A.* **1956**, *235*, 518-536.

[12] (a) G. Kresse, J. Hafner. *J. Phys. Rev. B: Condens. Matter.* **1993**, 47, 558–561; (b) G. Kresse, J. Hafner. *Phys. Rev. B: Condens. Matter*. **1994**, 49, 14251–14269; (c) G. Kresse, J. Furthmuller. *Comput. Mater. Sci.* **1996**, 6, 15–50; (d) G. Kresse, J. Furthmuller. *Phys. Rev. B: Condens. Matter.* **1996**, 54, 11169–11186.

[13] (a) P. E. Blöchl. *Phys. Rev. B: Condens. Matter* **1994**, 50, 17953–17979; (b) J. Hafner. *J. Comput. Chem.* **2008**, 29, 2044–2078.

[14] J. P. Perdew, J. A. Chevary, S. H. Vosko, K. A. Jackson, M. R. Pederson, D. J. Singh and C. Fiolhais, Phys. Rev. B: Condens. Matter 1992, 46, 6671–6687.

[15] S. L. Dudarev, G. A. Botton, S. Y. Savrasov, C. J. Humphreys, A. P. Sutton. *Phys. Rev. B* **1998**, 57, 1505–1509.

[16] A. Du, Y. H. Ng, N. J. Bell, Z. Zhu, R. Amal, S. C. Smith. *J. Phys. Chem. Lett.* **2011**, 2, 894–899.

[17] (a) A. D. Becke. *J. Chem. Phys.* **1993**, 98, 5648–5652; (b) C. Lee, W. Yang, R. G. Parr. *Phys. Rev. B* **1988**, 37, 785–789.

[18] M. J. Frisch et al., Gaussian09W, Revision C01; Gaussian, Inc.: Wallingford, CT, 2009.

[19] J. Tomasi, B. Mennucci, R. Cammi. *Chem. Rev.* **2005**, 105, 2999–3094.

[20] T. Lu, F. J. Chen. *Comp. Chem.* **2012**, 33, 580–592.
